# Chromatin conformation changes in peripheral blood can detect prostate cancer and stratify disease risk groups

**DOI:** 10.1186/s12967-021-02710-y

**Published:** 2021-01-28

**Authors:** Heba Alshaker, Robert Mills, Ewan Hunter, Matthew Salter, Aroul Ramadass, Benjamin Matthew Skinner, Willem Westra, Jayne Green, Alexandre Akoulitchev, Mathias Winkler, Dmitri Pchejetski

**Affiliations:** 1 School of Medicine, University of East Anglia, Norwich, UK; 2 Department of Urology, Norfolk and Norwich NHS Trust, Norwich, UK; 3 Oxford BioDynamics Limited, Oxford, UK; 4 School of Life Sciences, University of Essex, Wivenhow Park, Colchester, CO4 3SQ UK; 5 Department of Surgery and Cancer, Imperial College London, London, UK

**Keywords:** Prostate cancer, Diagnosis, Prognosis, Epigenetics, Nucleome, Blood test

## Abstract

**Background:**

Current diagnostic blood tests for prostate cancer (PCa) are unreliable for the early stage disease, resulting in numerous unnecessary prostate biopsies in men with benign disease and false reassurance of negative biopsies in men with PCa. Predicting the risk of PCa is pivotal for making an informed decision on treatment options as the 5-year survival rate in the low-risk group is more than 95% and most men would benefit from surveillance rather than active treatment. Three-dimensional genome architecture and chromosome structures undergo early changes during tumourigenesis both in tumour and in circulating cells and can serve as a disease biomarker.

**Methods:**

In this prospective study we screened whole blood of newly diagnosed, treatment naïve PCa patients (n = 140) and cancer-free controls (n = 96) for the presence of 14,241 chromosomal loops in the loci of 425 genes.

**Results:**

We have detected specific chromosome conformation changes in the loci of *ETS1, MAP3K14, SLC22A3* and *CASP2* genes in peripheral blood from PCa patients yielding PCa detection with 80% sensitivity and 80% specificity. Further analysis between PCa risk groups yielded prognostic validation sets consisting of *HSD3B2, VEGFC, APAF1, BMP6, ERG, MSR1, MUC1, ACAT1* and *DAPK1* genes that achieved 80% sensitivity and 93% specificity stratifying high-risk category 3 vs low risk category 1 and 84% sensitivity and 89% specificity stratifying high risk category 3 vs intermediate risk category 2 disease.

**Conclusions:**

Our results demonstrate specific chromosome conformations in the blood of PCa patients that allow PCa diagnosis and risk stratification with high sensitivity and specificity.

## Background

In the Western world prostate cancer (PCa) is now the most commonly diagnosed non-cutaneous cancer in men and is the second leading cause of cancer-related death [1, 2]. Many men as young as 30 show evidence of histological PCa [3, 4], most of which is microscopic and some may not manifest clinically. For the diagnosis and prognosis, prostate specific antigen (PSA), magnetic resonance imaging (MRI), an invasive needle biopsy, Gleason score and disease stage are used [5, 6].

The only available blood test for PCa in widespread clinical use involves measuring circulating levels of PSA (at 4 ng/ml has 21% sensitivity and 93% specificity, PCa prevention trial) [7], however, the prostate size, benign prostatic hyperplasia and prostatitis may also increase PSA levels. PSA cut off levels are age-specific. American urological association thresholds (ng/ml) are as follows: age 40–49 = 2.5; age 50–59 = 3.5; 60–69 = 4.5; 70–79 = 6.5. In the UK, in men aged 50–69 years, a reading of 3.0 ng/ml or higher leads to urgent referral for further investigation. However, men with low PSA can have cancer. In a large US study, 2950 men (age range, 62 to 91 years) with PSA < 4.0 ng/ml underwent biopsy. PCa was diagnosed in 449 (15.2%); 67 of these 449 cancers (14.9%) had a Gleason score of 7 or higher [8]. Furthermore, younger patients with Gleason 8–10 cancer and PSA levels of < 4.0 ng/ml have more aggressive disease than those with PSA levels of 4 to 9.9 ng/ml [9]. In early PCa, PSA testing is not specific enough to differentiate between early-stage invasive cancers and latent, non-lethal tumours that might otherwise have remained asymptomatic during a man’s lifetime. In advanced PCa, PSA kinetics are used as a clinical surrogate endpoint for outcome. However, while they do give a general prognosis they lack specificity for the individual [10]. A number of more specific blood tests are emerging for PCa detection including 4 K blood test (AUC 0.8) and PHI blood test (90% sensitivity, 17% specificity) [11]. Both PHI and 4 K tests have a proposed prognostic value [12], however, its utility is limited [13] and it is currently not widely used in clinical practice. PSA levels, clinical disease stage and Gleason score are used to establish the severity of PCa and stratify patients to risk groups [13]. To date, there is no prognostic blood test available that allows differentiation between low- and high-risk PCa.

Genome-wide association studies (GWAS) have identified more than 100 PCa susceptibility loci, explaining ∼30% of the familial risk for this disease [14]. However, most of PCas are not familial and are linked to unidentified multiple somatic mutations. These include mutations in p53 (up to 64% of tumours), p21 (up to 55%), p73 and MMAC1/PTEN tumour suppressor genes [15], as well as alterations in 25 PCa susceptibility loci [16], but these mutations do not explain all the observed effects on gene regulation. Indeed, one interesting observation that arose from GWAS, was that most of the loci within the genome that confer risk to diseases including cancer are located outside of known protein-coding regions [17].

Epigenetic modifications play an important role in PCa progression [18, 19]. Aberrant DNA methylation (hypo- and hypermethylation) is the best-characterized cancer-related epigenetic alteration in PCa [20, 21]. Histone modifications also contribute to PCa progression [22, 23]. We have previously found that in human cells epigenetic mechanisms involving dynamic and multi-layered chromosomal loop interactions are powerful regulators of gene expression [24]. Chromosome conformation capture (3C) technologies allow these signatures to be recorded and have gained considerable attention for disease diagnosis [25–28]. Prostate tumours have been shown to undergo long-range epigenetic alterations in 3-dimension chromosome conformations and distinct epigenetic signatures were found in circulating DNA from PCa patients [29]. Another study identified a strong association of single nucleotide polymorphism rs11672691 at 19q13 with clinical features indicative of aggressive PCa. Mechanistically, this locus resides in an enhancer element and alters the binding site of HOXA2, a novel oncogenic transcription factor [30].

**Fig. 1:**
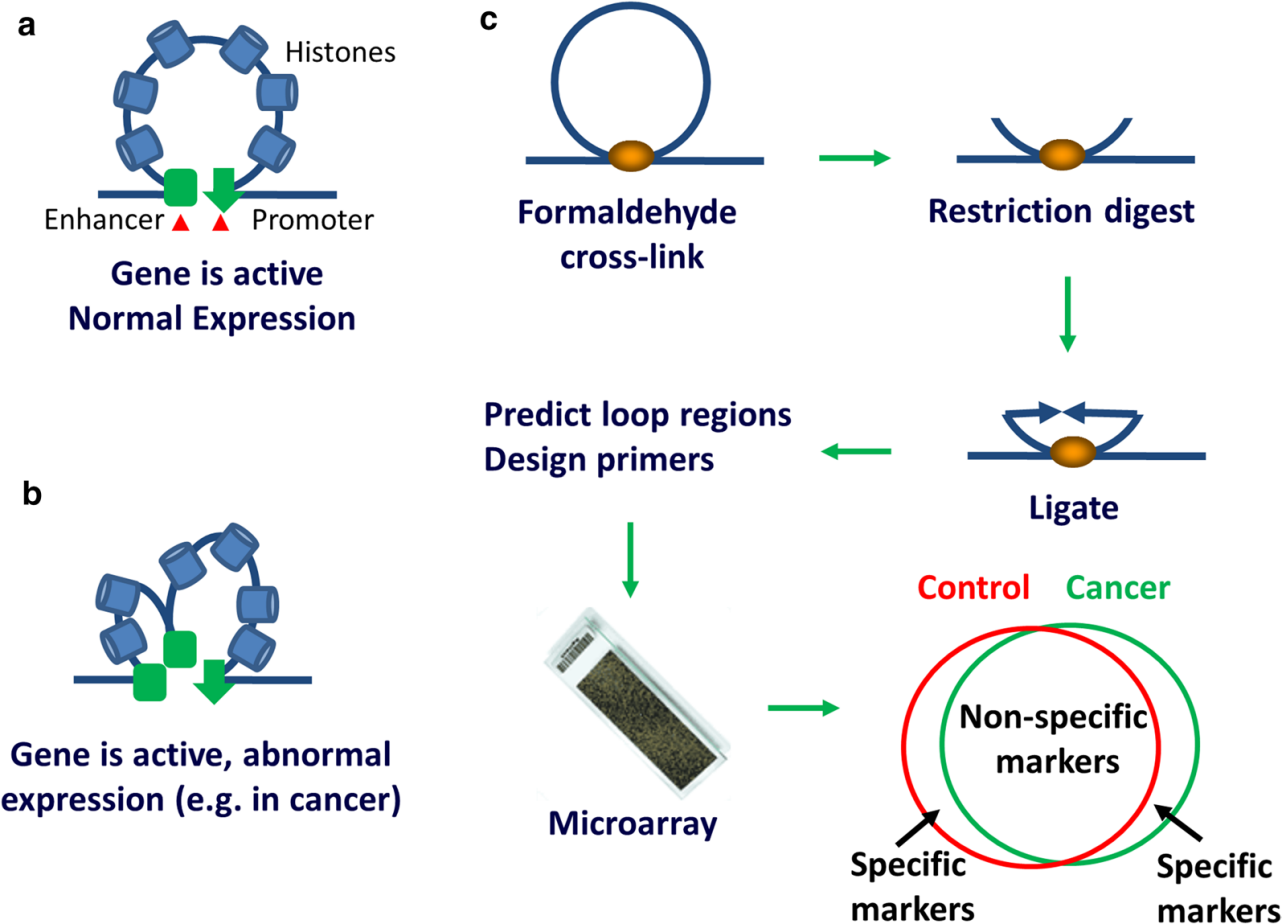
Chromosomal loops structure and conformation assay. **a **Chromosomal loop contains the enhancer region that increases target gene promoter activity. **b** During cancer progression there is increased looping in some tumour-related genes leading to abnormal gene expression. **c** Chromosome conformation assay: DNA is crosslinked, digested, ligated and new sequences (in places where loops were) are predicted using relevance machine vector. Loops presence is then detected using DNA microarray. Resulting markers are analysed using multivariate analysis yielding specific epigenetic signatures for selected patient cohorts

We have developed a novel epigenetic assay, as a next generation of the 3C technique [31]. EpiSwitch™ technology employs 3C technique and algorithmic-based analysis to identify a panel of epigenetic differences capable of distinguishing between cancer-free controls and diseased tissue samples. It detects epigenetic regulatory signature changes in the higher order structures of human chromosomes at the loci implicated in the onset and progression of the disease. It offers highly effective means of screening; early detection; monitoring and prognostic analysis of major diseases associated with aberrant gene expression [24]. One of the main advantages of using 3C-based chromatin interactions as biomarkers is that DNA cross-linking is relatively stable, and following proximity ligation, give rise to a stable DNA product (Fig. 1) [32]. Using EpiSwitch™ technology, we have shown the presence of melanoma-specific chromatin conformations in peripheral blood mononuclear cells (PBMCs) and primary tumours of melanoma patients [33, 34]. Fractionation studies showed that the detected signature comes from lymphocytes and not circulating tumour cells [33, 34]. The concentration of circulating tumour cells in circulation is too low to allow detection of chromosomal conformations, while circulating free DNA does not retain 3D conformations [33, 34]. In this study, we used the EpiSwitch™ assay to screen for, define and evaluate specific chromosome conformations in the blood of PCa patients and to identify loci with potential to act as diagnostic and prognostic markers.

## Methods

### Patient population

A total of 140 PCa patients and 96 cancer-free controls were recruited, in two cohorts. Cohort 1: men with (n = 105) or without (n = 77) PCa diagnosis attending urology clinic at Imperial College Healthcare NHS Trust were prospectively recruited from October 2010 through September 2013. Cohort 2: Patients' samples (19 cancer-free controls and 35 PCa) obtained from Janssen (USA). Upon recruitment, a single blood sample (5 ml) was collected from PCa patients using the current practice for needle and blood collection methods into the BD Vacutainer^®^ plastic EDTA tubes. Blood samples were passively frozen and stored at − 80 °C until processed. Prostate tumour samples were obtained from previously recruited patients (n = 5) that subsequently underwent radical prostatectomy at Imperial College Healthcare NHS Trust. Patient clinical characteristics are shown in Additional file 1: Table S1. The primary endpoint of this study was to detect changes in chromosomal conformations in PBMCs from PCa patients in comparison to cancer-free controls. Therefore, all treatment naïve PCa patients were eligible for this study irrespective of grade, stage and PSA levels. Patients that had previous chemotherapy or patients with other cancers were excluded from this study. PCa diagnosis was established as per clinical routine and patients were assigned to appropriate treatment. For prognostic study (secondary endpoint), patients were stratified according to the relevant National Comprehensive Cancer Network (NCCN) risk groups (Additional file 1: Table S2). No follow up study was conducted. All patients who participated in the trial have given informed consent. The study was approved by the local ethics committee (Imperial College NHS Trust REF 07/MRE09/54). All procedures and protocols were performed in accordance with the relevant guidelines and regulations. Based on the preliminary findings in melanoma [33, 34], an a priori power analysis was performed using the pwr.t.test function in the R package pwd.

### Stepwise diagnostic and prognostic biomarker discovery process using EpiSwitch™ technology and data analysis

EpiSwitch™ technology platform (Oxford Biodynamics, Oxford, UK) pairs high resolution 3C results with regression analysis and a machine learning algorithm to develop disease classifications [33–36]. To select epigenetic biomarkers that can diagnose cancers, samples from patients suffering from cancer, in comparison to cancer-free control samples were screened for statistically significant differences in conditional and stable profiles of genome architecture (Fig. 1). All the samples used for the nested polymerase chain reaction (PCR) diagnostic and prognostic biomarker discovery were processed as per the manufacturer’s instructions using the proprietary EpiSwitch™ reagents and standard protocols (the standard protocol for nested PCR is Work Instruction 52 (WI52) and for real-time quantitative PCR (qPCR) is WI82 [36]). The samples for the development of the qPCR process were processed using the EpiSwitch™ reagents and protocols as described previously [36]. Briefly, 50 µl of whole blood was suspended in 450 µl PBS, formaldehyde containing solution was added and the mixture incubated at room temperature for 15 min to fix the samples. Glycine solution was added to bind any unreacted formaldehyde and quench the reaction. Cell membrane lysis was performed, and the intact nuclei purified using a density cushion centrifugation. TaqI restriction enzyme digestion (Fermentas FastDigest™ TaqI kit) was performed and proximity ligation with T4 DNA ligase (Takara T4 Ligase kit) was used to capture the inter/intra chromatin associations as chromosome conformation signatures. Proteinase K (Sigma) was added to the libraries to digest the constraining protein content of the samples and allow for greater accessibility in the PCR. The Proteinase K was denatured, and cross-links partially reversed at 90 °C. The protocols were repeated to produce the required volume of library for nested PCR investigation. The same process was applied to the matched tissue samples with the addition of an initial incubation in 0.125% (v/v) Collagenase (Sigma) for 37 °C with gentle agitation for 30 min until the tissue was suspended. The freed cells were pelleted by centrifugation at 800*g* for 5 min and the supernatant removed before being resuspended in 500 µl PBS and subjected to the same protocol as described previously [37]. In short, the assay was performed on a whole blood sample by first fixing chromatin with formaldehyde to capture intrachromatin associations, as described before [37]. The fixed chromatin was then digested into fragments with TaqI restriction enzyme, and the DNA strands were joined favouring cross-linked fragments. The cross-links were reversed and PCR performed using the primers previously established by the EpiSwitch™ software (Fig. 1).

The version 2 EpiSwitch™ reagents and protocols were used to generate the libraries for the qPCR translation stage. These follow the protocol as described previously [37] with an additional detergent-based step between the density cushion centrifugation and TaqI restriction to increase enzyme accessibility and restriction rates. In addition, the Proteinase K treatment was replaced with the use of the QIAmp^®^ DNA FFPE Tissue Kit (Qiagen), starting at the step of resuspension of sample in ATL buffer.

**Fig. 2:**
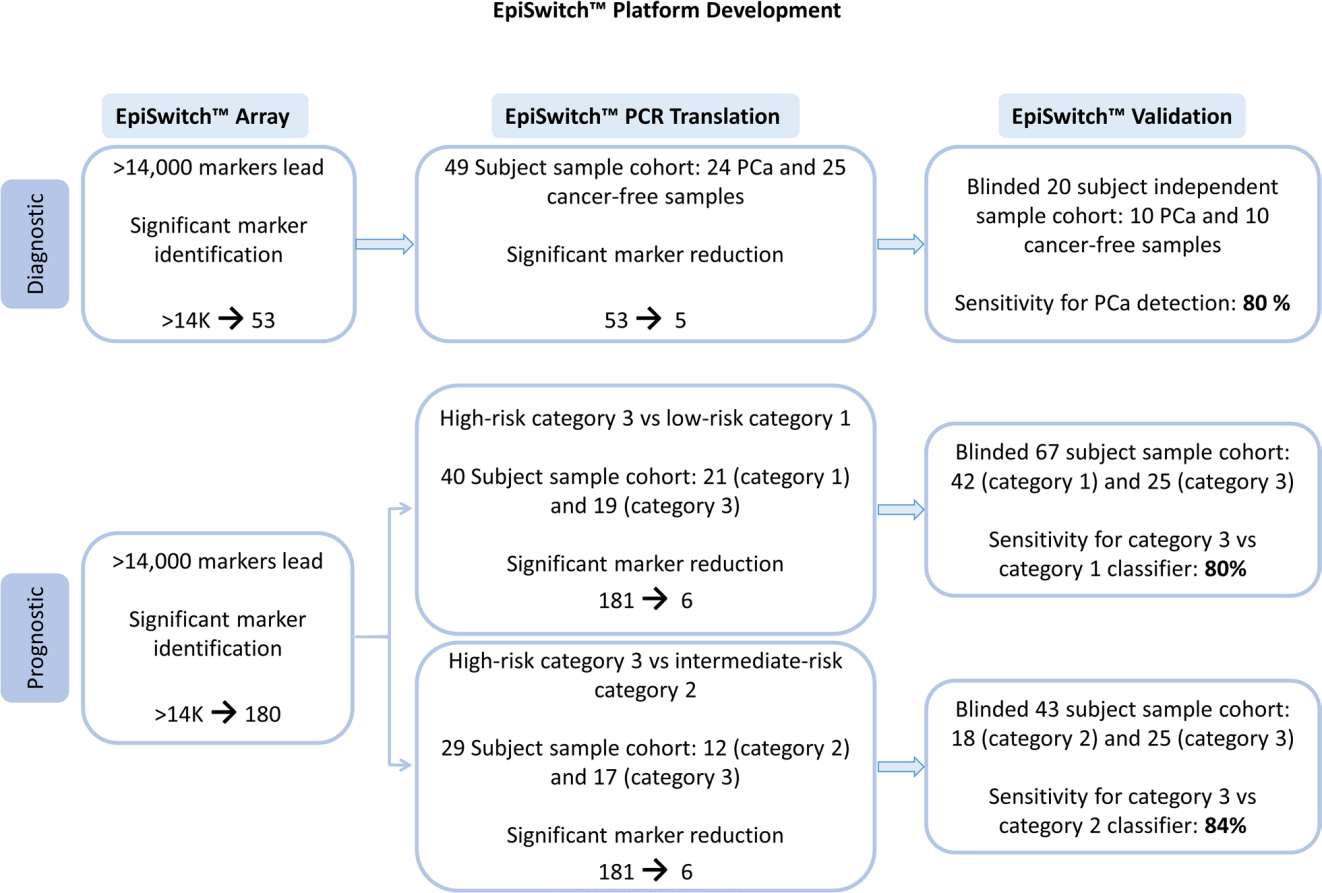
Three-step approach to identify, evaluate, and validate diagnostic and prognostic biomarkers for prostate cancer (PCa)

EpiSwitch™ was used on blood samples in a three-step process to identify, evaluate, and validate statistically significant differences in chromosomal conformations between PCa patients and cancer-free controls (Fig. 2). For the first step, sequences from 425 manually curated PCa-related genes (obtained from the public databases (www.ensembl.org)) were used as templates for this computational probabilistic identification of regulatory signals involved in chromatin interaction (Additional file 1: Table S3). A customized CGH Agilent microarray (8 × 60 k) platform was designed to test technical and biological repeats for 14,241 potential chromosome conformations across 425 genetic loci. Eight PCa and eight cancer-free control samples were competitively hybridized to the array, and differential presence or absence of each chromosome conformation was defined by LIMMA linear modelling with empirical Bayes moderation of the standard errors, subsequent abundance filtering and cluster analysis. This initially revealed 60 chromosomal interactions with the ability to best discriminate PCa patients from cancer-free controls (Fig. 2). For the second evaluation stage, the 60 chromosomal interactions were translated into EpiSwitch™ PCR based-detection assays. Nested PCR primers were designed for the 60 chromosomal interactions and these tested using a pooled PCa and a pooled cancer-free control, to ascertain if they produced the expected PCR product. Of these 60 primer combinations, 7 failed to produce PCR products and were subsequently dropped from further investigation. The 53 successful primer combinations were screened on PCa and cancer-free controls (n = 6 in each group). Feature reduction of the 53 PCR was performed using an univariant statistical approach (Fishers Exact test), this analysis identified 15 nested PCR markers with the most statistical discernibility between PCa and cancer-free controls.

The known sample cohort used to identify the 15 markers was expanded to include 49 samples (PCa n = 24, cancer-free controls n = 25) to provide better statistical power for marker selection. With the expanded cohort univariate (Fishers Exact test) and multivariant (GLMNET, penalized logistic regression, alpha 1 (lasso) and lambda 0.01) statistical approaches were used to further identify markers with the greatest statistical discernibility. GLMNET was used in combination with permutation, where the cohort was split 500 times randomly into a training (0.667) and test (0.333). A GLMNET model is developed for each data split (500) and the markers used in the model are given a vote, the frequency of use of the marker is tallied, markers used in all data splits would get be voted 500 times. This resampling methodology counters false positives in terms of marker selection. In combination with the univariate test for independence (Fishers Exact test), the final model identified was made up of a five-marker signature (Table 1). Principal component analysis was also used to determine group variance and to identify potential outlier samples. The 49 known samples used for marker reduction were used to create a logistic model classifier with five-fold cross validation. The five markers were further statistically assessed using 500 randomised classifiers, 250 logistic model and 250 naïve bayes classifiers with data resampling in each iteration.

**Table 1:** Five-marker signature used for the diagnosis of prostate cancer (PCa)

Markers	Gene symbol	Gene name	P value
PCa.57.59	*ETS1*	*ETS proto-oncogene 1, transcription factor*	0.11
PCa.81.83	*MAP3K14*	*Mitogen-activated protein kinase kinase kinase 14*	0.11
PCa.73.75	*SLC22A3*	*Solute carrier family 22 member 3*	0.107
PCa.77.79	*SLC22A3*	*Solute carrier family 22 member 3*	0.005
PCa.189.191	*CASP2*	*Caspase 2*	0.137

For the last step, to further validate the chromosome conformation signature used to inform PCa diagnosis, the training sample cohort size was expanded to 95 PCa and 96 cancer-free control samples. The five-marker set was tested on a blinded, independent (n = 20) cohort of blood samples (Additional file 1: Table S4). The results were analysed using Bayesian Logistic modelling, p-value null hypothesis (Pr(N|z|) analysis, Fisher-Exact P test and Glmnet**.**

Data analysis and presentation were performed in accordance with CONSORT recommendations. All measurements were performed in a blinded manner. STARD criteria have been used to validate the analytical procedures. A similar stepwise approach was followed for the identification of prognostic markers. A subset of prognostic markers was translated from the nested PCR format to qPCR assays with hydrolysable probes, to the industry standards of MIQE-compliant qPCR. The alternative qPCR format assays were validated on a subset of the prognostic sample cohort.

### Nested polymerase chain reaction (PCR)

Sequence specific oligonucleotides were designed around the chosen sites for screening potential markers by nested PCR using Primer3. All PCR amplified samples were visualized by electrophoresis in the LabChip GX, using the LabChip DNA 1 K Version2 kit (Perkin Elmer, Beaconsfield, UK) and internal DNA marker was loaded on the DNA chip according to the manufacturer’s protocol using fluorescent dyes. Fluorescence was detected by laser and electropherogram read-outs translated into a simulated band on gel picture using the instrument software. The threshold we set for a band to be deemed positive was 30 fluorescence units and above.

### Real-time quantitative PCR (qPCR) assay development

Both the inner and outer primer combinations of primers for markers identified in the nested PCR investigation were subjected to a temperature gradient PCR to identify the optimum annealing temperature using EpiSwitch™ libraries as the template inputs. The PCR products were detected using SYBR green amplification and melt curves. All PCR amplified samples were visualised by electrophoresis. The PCR products were gel purified, sequenced and mapped to the human reference genome to validate the 3C products. The sequence verified PCR products were used to create standards for absolute quantification starting at a 10^6^ copies standard. Hydrolysis probes were designed across the restriction/ligation junction of the 3C products to produce specific detection of the 3C products of interest. The hydrolysis probe detection was again subjected to a temperature gradient PCR to identify the optimum annealing/extension temperature for the specific detection of the PCR products. The generated 10^6^ copy standard was used as the positive control alongside standard EpiSwitch template and no template controls.

### Detection of similar epigenetic markers in blood and matching primary prostate tumours

Primary tumour samples were obtained from biopsies of selected patients from Imperial College Healthcare NHS Trust cohort (n = 5). The pulverized tissue samples were incubated in 0.125% collagenase at 37 °C with gentle agitation for 30 min. The resuspended cells (250 µl) were then centrifuged at 800*g* for 5 min at room temperature in a fixed arm centrifuge, supernatant removed, and the pellets resuspended in phosphate-buffered saline. Primary tumours and matching blood samples were analysed for the presence of the six-marker set for categories 3 vs 1 and 3 vs 2 at a fixed range of assay sensitivity (dilution factor 1:2). When matching PCR bands of the correct size were detected, a score of 1 was assigned, detection of no band was assigned a score of 0 (Table 2).

**Table 2:** Detection of similar epigenetic markers in blood and in matching primary prostate tumours at a fixed range of assay sensitivity

Markers for category	Gene location	Category	Blood samples						Tissue samples						Total number of common positive markers	Concordance between blood and tissue samples (%)
			1				3	Total number of positive markers in blood samples	1				3	Total number of positive markers in tissue samples		
		Patient	A	B	C	D	E		A	B	C	D	E			
		Markers														
3 vs 1	BMP6	PCa.119.37.39	1	1	0	1	1	4	1	1	1	0	1	4	3	60
	ERG	PCa.119.65.67	1	1	1	1	1	5	1	0	1	0	1	3	3	60
	MSR1	PCa.119.77.79	1	0	0	0	1	2	0	0	1	0	1	2	1	20
Common	MUC1	PCa.119.121.123	1	1	1	1	0	4	1	1	1	1	1	5	4	80
	DAPK1	PCa.119.165.167	1	0	1	1	0	3	0	0	1	0	1	2	1	20
	ACAT1	PCa.119.57.59	1	1	1	1	1	5	1	1	1	1	1	5	5	100
3 vs 2	HSD3B2	PCa.119.129.131	1	1	0	1	1	4	1	1	1	0	1	4	3	60
	VEGFC	PCa.119.205.207	1	1	1	1	1	5	1	1	1	1	1	5	5	100
	APAF1	PCa.119.49.51	1	1	1	1	1	5	0	1	1	1	1	4	4	80

When a PCR band of the correct size is detected, it is given a score of 1. When no band is detected, it is given a score of 0

ACAT1: acetyl-CoA acetyltransferase 1; APAF1: apoptotic peptidase activating factor 1; BMP6: bone morphogenetic protein 6; DAPK1: death associated protein kinase 1; ERG: ETS transcription factor ERG; HSD3B2: hydroxy-delta-5-steroid dehydrogenase, 3 beta- and steroid delta-isomerase 2; MSR1: macrophage scavenger receptor 1; MUC1: mucin 1, cell surface associated; VEGFC: vascular endothelial growth factor C

## Results

### Identification of the diagnostic markers

**Fig. 3:**
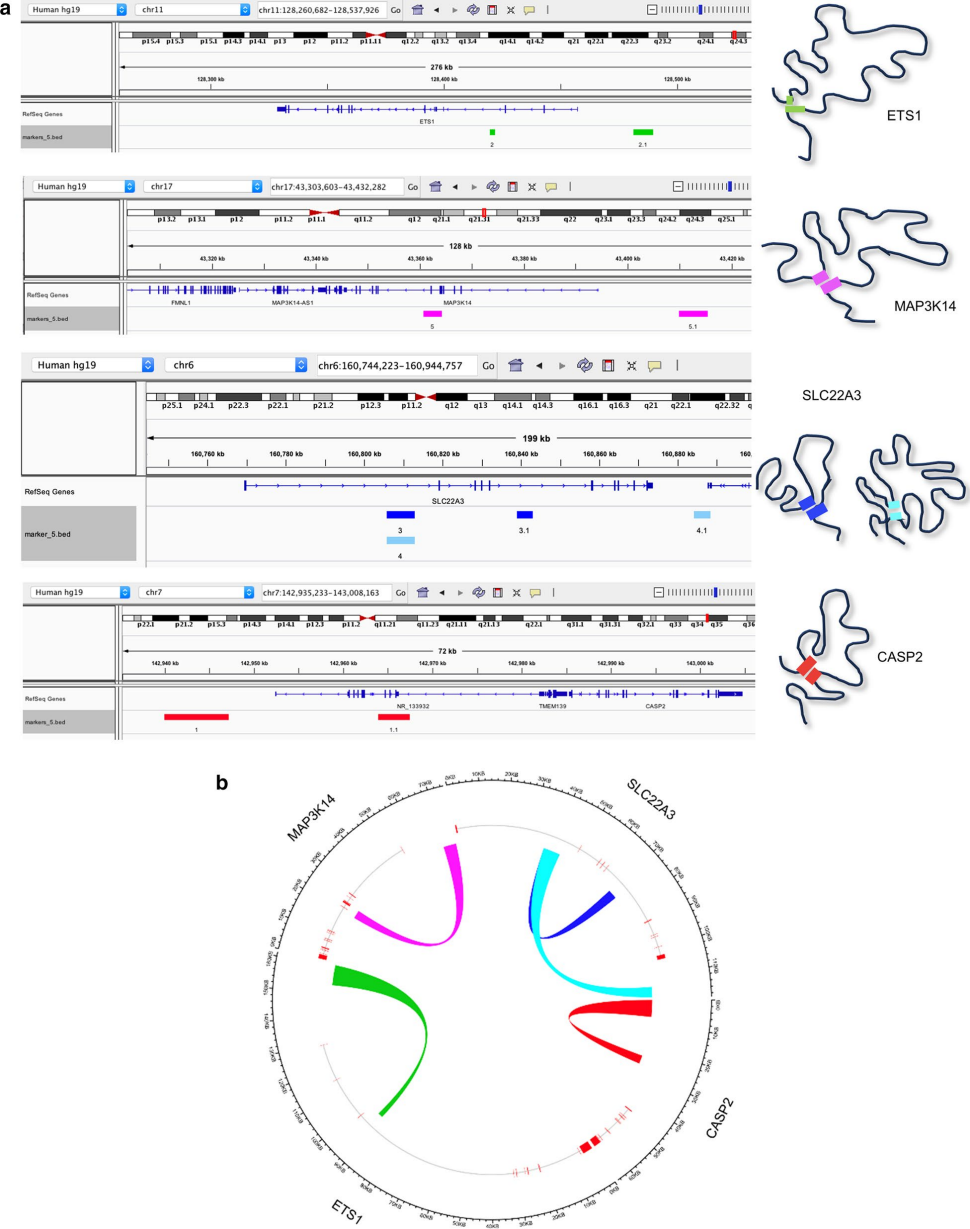
Graphical representation of the genomic co-ordinates of the ETS1, MAP3K14, SLC22A3 and CASP2 chromosome conformation signature markers associated with PCa.** a** The Ensembl browser view of the *ETS1, MAP3K14, SLC22A3 and CASP2* genes on chromosomes 11, 17, 6 and 7 with the EpiSwitch™ sites marked with green, pink, blue and red symbols, respectively. **b** Circos plots of ETS1 (green) MAP3K14 (pink), SLC22A3 (blue) and CASP2 (red) chromosome conformation signature markers showing the chromosomal loop

We have applied a stepwise diagnostic biomarker discovery process using EpiSwitch™ technology as described in methods. A customized CGH Agilent microarray (8 × 60 k) platform was designed to test technical and biological repeats for 14,241 potential chromosome conformations across 425 genetic loci (Additional file 1: Table S3) in eight PCa and eight cancer-free control samples (Figs. 1, 2). The presence or absence of each locus was defined by LIMMA linear modelling, subsequent binary filtering and cluster analysis. In the second evaluation stage, nested PCR was used for the 53 selected biomarkers further reducing them to 15 markers and finally to a five-marker signature (Fig. 2). This distinct chromosome conformation disease signature for PCa comprised of chromosomal interactions in five genomic loci: *ETS proto-oncogene 1, transcription factor* (*ETS1*)*, mitogen-activated protein kinase kinase kinase 14* (*MAP3K14*)*, solute carrier family 22 member 3* (*SLC22A3*) *and caspase 2* (*CASP2*) (Table 1). The presence of an altered loop in each of the genes individually (except SLC22A3) was not statistically significant in predicting cancer. Only a combination of five of them provided required accuracy in the logistic model (please see the Stepwise Data Analysis section in Methods). The genomic locations of specific chromosomal loops in *ETS1, MAP3K14, SLC22A3* and *CASP2* genes in the chromosome conformation signature (Table 1) were mapped on their relative chromosomes in Fig. 3. The two genomic sites that corresponded to the junction of each chromosome conformation signature locus for *ETS1, MAP3K14, SLC22A3 and CASP2* genes were mapped on chromosome 11 from 128,260,682 to 128,537,926; chromosome 17 from 43,303,603 to 43,432,282; chromosome 6 from 160,744,223 to 160,944,757 and chromosome 7 from 142,935,233 to 143,008,163, respectively, with the EpiSwitch™ sites marked with colour (Fig. 3a). Circos plots of *ETS1, MAP3K14, SLC22A3* and *CASP2* chromosome conformation signature markers showing the chromosomal loops are depicted in Fig. 3b.

**Fig. 4:**
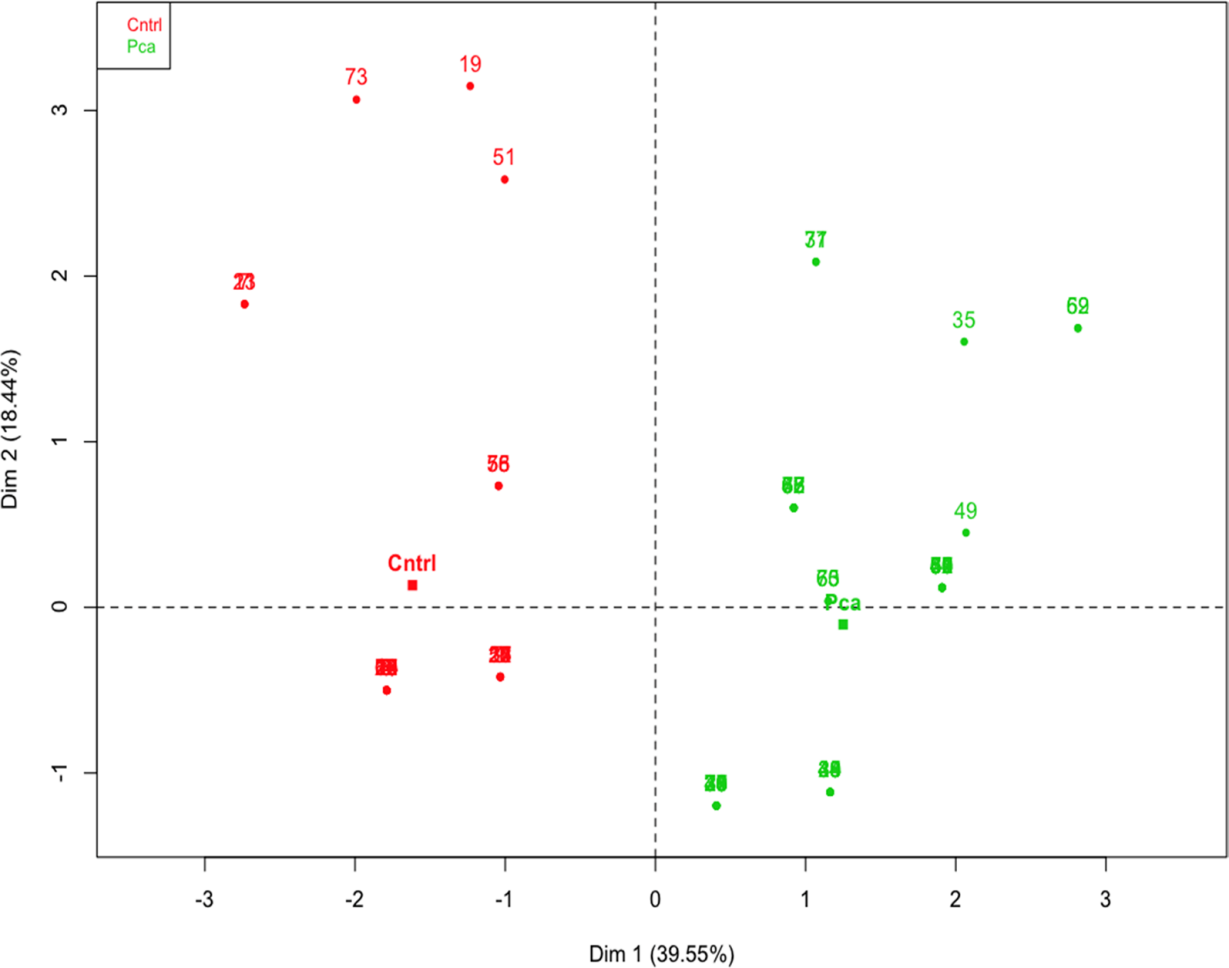
Principal component analysis for the five-markers applied to 78 samples containing two groups. First group, 49 known samples (24 PCa and 25 cancer-free controls (Cntrl)) combined with a second group of 29 samples including, 24 PCa samples and 5 healthy Cntrl samples. PCa samples in green and cancer-free Cntrl samples in red

Principal component analysis for the five-markers was used to determine group variance and to identify potential outlier samples. This analysis was applied to 78 samples containing two groups. First group, 49 known samples (24 PCa and 25 cancer-free controls) combined with a second group of 29 samples including, 24 PCa samples and 5 cancer-free control samples demonstrating no outliers (Fig. 4). The final training set was built using 95 PCa and 96 cancer-free control samples and then tested on an independent blinded validation cohort of 20 samples (10 cancer-free controls and 10 PCa). The sensitivity and specificity for PCa detection using chromosomal interactions in five genomic loci were 80% (CI 44.39% to 97.48%) and 80% (CI 44.39% to 97.48%), respectively (Additional file 1: Table S4).

### Identification of the prognostic markers

To select epigenetic biomarkers that can stratify PCa, the samples from PCa patients categorised into risk group categories 1–3 (low (n = 42), intermediate (n = 18) and high (n = 25), respectively, Additional file 1: Table S2) were screened for statistically significant differences in conditional and stable profiles of genome architecture. EpiSwitch™ was used on blood samples in a three-step process to identify, evaluate, and validate statistically significant differences in chromosomal conformations between PCa patients at different stages of the disease (Fig. 2). For the first step, the array used covered 425 genetic loci, with testing probes for the total of 14,241 potential chromosomal conformations. Patients with high-risk PCa category 3 were compared to low-risk category 1 or intermediate-risk category 2. In total, 181 potential stratification marker leads for PCR evaluation were identified using enrichment statistics (Additional file 1: Table S5). The top 70 top markers were then taken to the next stage of PCR detection for further evaluation and stratification of high-risk category 3, vs low-risk category 1 patient samples. The 70 markers were reduced to 16 and finally a six-marker set for high category 3 vs low category 1 was established (Additional file 1: Table S6). The best markers were identified using Chi-square and then built into a classifier on a testing set of category 1 (n = 21) and category 3 (n = 19). An independent cohort of category 1 (n = 21) and category 3 (n = 6) which were not used for any marker reduction were then used for first round of blind validation. Similarly, a six-marker set was evaluated for high-risk category 3 vs intermediate-risk category 2 on a testing set of category 3 and category 2 including, 17 and 12 samples, respectively. An independent cohort of category 2 and category 3 (n = 6 in each group) which were not used for any marker reduction were then used for first round of blind validation.

For the last step, to further validate the chromosome conformation signature used to inform PCa prognosis, the six-marker set for high-risk category 3 vs low-risk category 1 was tested on a larger, more representative cohort. The original blind cohort was expanded to 67 samples, including 40 samples used in marker reduction (Additional file 1: Table S7). Similarly, the six-marker set for high-risk category 3 vs intermediate-risk category 2 was tested on a on a larger, more representative cohort. The original blind cohort was expanded to 43 samples (Additional file 1: Table S8).

A six-marker set for category 3 vs category 1 was established. This set contained bone morphogenetic protein 6 (*BMP6), ETS transcription factor ERG* (*ERG*), macrophage scavenger receptor 1 (*MSR1*)*, mucin 1* (*MUC1*)*, acetyl-CoA acetyltransferase 1* (*ACAT1*) and *death-associated protein kinase 1* (*DAPK1*) genes (Additional file 1: Table S6). Six-biomarkers were identified for high-risk category 3 vs intermediate-risk category 2, including *hydroxy-delta-5-steroid dehydrogenase, 3 beta- and steroid delta-isomerase 2* (*HSD3B2), vascular endothelial growth factor C* (*VEGFC*)*, apoptotic peptidase activating factor 1* (*APAF1*)*, MUC1, ACAT1* and *DAPK1*. Notably, the last three-biomarkers (*MUC1, ACAT1* and *DAPK1*) were common between categories 1 vs 3 and 3 vs 2 (Additional file 1: Table S6). Stratification of high-risk category 3 vs low-risk category 1 PCa using chromosomal interactions in six genomic loci showed sensitivity of 80% (CI 59.30% to 93.17%) and specificity of 93% (CI 80.52% to 98.50%) in the blind cohort of 67 samples (Additional file 1: Table S7). Similarly, the six-marker set for high-risk category 3 vs intermediate-risk category 2 was tested on a on a larger, more representative cohort of 43 samples demonstrating sensitivity of 84% (CI 63.92% to 95.46%), and specificity of 89% (CI 65.29% to 98.62%) (Additional file 1: Table S8).

### Detection of similar epigenetic markers in blood and matching primary prostate tumours

Using five matching peripheral blood and primary tumour samples, we have compared the epigenetic markers identified in peripheral circulation (Additional file 1: Table S6) to the tumour tissue. Our results showed that a number of deregulation markers detected in the blood as part of stratifying signatures for category 1 vs 3 and category 2 vs 3 could be detected in the tumour tissue (Table 2). This demonstrates that the chromosome interactions that can be detected systemically could be detected under same conditions in the primary site of tumourigenesis.

### Real-time quantitative PCR (qPCR) translation

The 16 statistically significant markers identified in the prognostic marker screen were translated to real-time qPCR assays. Due to the nature of 3C templates the sequence available for suitable primer and probe design for qPCR is constrained. This led to 6 of the markers failing to be translated to the qPCR assay. The assays were further assessed over the nested PCR assays by screening a 40-sample subset of the prognostic sample cohort. The cohort consisted of 10 cancer-free controls, 10 category 1, 10 category 2 and 10 category 3 samples. A new classifier was produced using the qPCR data and tested with resampling for the ability to distinguish between the cancer-free controls and PCa based on 6 markers, demonstrating average sensitivity of an 91% and specificity of 89.3% based on 500 randomisations for cross validation and a 66/34% train/test split.

## Discussion

Timely diagnosis of PCa is crucial to reducing mortality. The European randomised study of screening for PCa has shown significant reduction in PCa mortality in men who underwent routine PSA screening [38, 39]. This notion was, however, not supported by the results of prostate, lung, colorectal, and ovarian (PLCO) cancer screening trial in the USA [40]. Total population screening leads to overdiagnosis of clinically insignificant disease and new less invasive tests capable of discriminating low- from high-risk disease are urgently required.

Our epigenetic analysis approach provides a potentially powerful means to address this need. The binary nature of the test (the chromosomal loop is either present or not) and the enormous combinatorial power (> 10^10^ combinations are possible with ~ 50,000 loops screened) may allow creating signatures that accurately fit clinically well-defined criteria. In PCa that would be discerning low-risk vs high-risk disease or identifying small but aggressive tumours and determining most appropriate therapeutic options. In addition, epigenetic changes are known to manifest early in tumourigenesis, making them useful for both diagnosis and prognosis [41].

In this study, we identified and validated chromosome conformations as distinctive biomarkers for a non-invasive blood-based epigenetic signature for PCa. Our data demonstrate the presence of stable chromatin loops in the loci of *ETS1, MAP3K14, SLC22A3 and CASP2* genes present only in PCa patients (Table 1). Validation of these markers in an independent set of 20 blinded samples showed 80% sensitivity and 80% specificity (Additional file 1: Table S4), which is remarkable for a PCa blood test. Interestingly, the expression of some of these genes has already been linked to cancer pathophysiology. ETS1 is a member of ETS transcription factor family. ETS1‐overexpressing prostate tumours are associated with increased cell migration, invasion and induction of epithelial‐to‐mesenchymal transition [42]. MAP3K14 (also known as nuclear factor-kappa-beta (NF-kβ)-inducing kinase (NIK)) is a member of MAP3K group (or MEKK). Physiologically, MAP3K14/NIK can activate noncanonical NF-kβ signalling and induce canonical NF-kβ signalling, particularly when MAP3K14/NIK is overexpressed [43]. A novel role for MAP3K14/NIK in regulating mitochondrial dynamics to promote tumour cell invasion has been described [44]. SLC22A3 (also known as organic cation transporter 3) is a member of SLC group of membrane transport proteins. SLC22A3 expression is associated with PCa progression [45]. CASP2 is a member of caspase activation and recruitment domains group. Physiologically, CASP2 can act as an endogenous repressor of autophagy [46]. Two of the identified genes (SLC22A3 and CASP2) were previously shown to be inversely correlated with cancer progression [45, 47, 48]. Importantly, the presence of the chromatin loop can have indeterminate effect on gene expression.

To screen for PCa prognostic markers we performed the EpiSwitch™ custom array to analyse competitive hybridization of DNA from peripheral blood from patients with low-risk PCa (category 1) and high risk PCa (category 3). Six-marker set was identified for high-risk category 3 vs low-risk category 1, including *BMP6, ERG, MSR1, MUC1, ACAT1* and *DAPK1*. Six-biomarkers were identified for high-risk category 3 vs intermediate-risk category 2, including *HSD3B2, VEGFC, APAF1, MUC1, ACAT1* and *DAPK1*. Three of these biomarkers (*MUC1, ACAT1* and *DAPK1*) were shared between these sets. Our data show high concordance between chromosomal conformations in the primary tumour and in the blood of matched PCa patients at stages 1 and 3 (Table 2). The prognostic significance and diagnostic value of some of these genes have previously been suggested [49–52]. *BMP6* plays an important role in PCa bone metastasis [50]. In addition to *ETS1, ERG* is another member of the ETS family of transcription factors. Overwhelming evidence, reviewed in [49], suggested that *ERG* is implicated in several processes relevant to PCa progression including metastasis, epithelial–mesenchymal transition, epigenetic reprogramming, and inflammation [49]. *MSR1* may confer a moderate risk for PCa [53]. *MUC1* is a membrane-bound glycoprotein that belongs to the mucin family. *MUC1* high expression in advanced PCa is associated with adverse clinicopathological tumour features and poor outcomes [51]. *ACAT1* expression is elevated in high-grade and advanced PCa and acts as an indicator of reduced biochemical recurrence-free survival [52]. *DAPK1* could function either as a tumour suppressor or as an oncogenic molecule in different cellular context [54]. *HSD3B2* plays a crucial role in steroid hormone biosynthesis and it is up-regulated in a relevant fraction of PCa that are characterized by an adverse tumour phenotype, increased androgen receptor signalling and early biochemical recurrence. [55]. *VEGFC* is a member of VEGF family and its increased expression is associated with lymph node metastasis in PCa specimens [56]. In a comprehensive biochemical approach, *APAF1* has been described as the core of the apoptosome [57].

Despite the identification of these loci, the mechanism of cancer-related epigenetic changes in PBMCs remains unidentified. The interaction, however, could be detected systemically and under same conditions in the primary site of tumourigenesis (Table 2). It is therefore assumed that the acquired changes must be directed by an external factor; presumably generated by the tumour cells. It is known that a significant proportion of chromosomal conformations are controlled by non-coding RNAs, which regulate the tumour-specific conformations [58, 59]. Tumour cells have been shown to secrete non-coding RNAs that are endocytosed by neighbouring or circulating cells and may change their chromosomal conformations [59, 60], and are possible regulators in this case.

RNA detection as a biomarker remains highly challenging (low stability, background drift, continuous variable for statistical stratification analysis). Chromosome conformation signatures offer well recognized stable binary advantages for the biomarker targeting use [25], specifically when tested in the nuclei [31], since the circulating DNA present in plasma does not retain 3D conformational topological structures present in the intact cellular nuclei. It is important to mention, that looking at one genetic locus does not equate to looking at one marker, as there may be multiple chromosome conformations present, representing parallel pathways of epigenetic regulation over the locus of interest.

Other technologies for plasma-based cancer detection such as using plasma cell-free DNA (cfDNA) methylomes [61] were recently introduced. The validity of this assay was tested to identify patients with renal cell carcinoma using urine cfDNA with area under the ROC curve (AUROC) of 0.86 [62]. It is worth noting that cfDNA is capturing post-apoptotic and necrotic passive distribution of free DNA, with significant variations, while EpiSwitch is measuring 3D genomic profiling in intact cells, capturing systemic surrogate readouts, which, for epigenetic modalities, have been shown to contain synchronized modulation at specific genetic loci concordant with primary sites of deregulation [63]. Systemic surrogate signatures at selective loci at the level of 3D genomics are sustained through exosome signalling and are not restricted to oncology [36].

One of the key challenges in the present clinical practice of PCa diagnosis is the time it takes to make a definitive diagnosis. So far, there is no single, definitive test for PCa. High levels of PSA will set the patient on a long journey of uncertainty where he will undergo a MRI scan followed by biopsy, if needed. Although a biopsy is more reliable than a PSA test, it is an invasive procedure at which targeting the cancer remains a significant issue. The five-set biomarker panel described here is based on a relatively inexpensive and well-established molecular biology technique (PCR). The samples are based on biofluid, which is simple to collect and provides clinicians with rapidly available clinical readouts within few hours. This in turn, offers a substantial time and cost savings and aids an informative diagnostic decision with the promise to help fill the gap in the current protocols for assertive diagnosis of PCa.

Predicting the risk of PCa is pivotal for making an informed decision on treatment options. Five-year survival rate in the low risk group is more than 95% and most men would benefit from less invasive therapy. Currently, PCa risk stratification is based on combined assessment of circulating PSA, tumour grade (from biopsy) and tumour stage (from imaging findings). The ability to derive similar information using a simple blood test would allow significant reduction in costs and would speed up the diagnostic process. Of particular importance in PCa treatment is identifying the few tumours that initially present as low-risk, but then progress to high-risk. This subset would therefore benefit from a quicker and more-radical intervention.

This study has several limitations including smaller number of patients in diagnostic validation cohort, unavailability of other clinical indices like PHI and 4 K (which are not part of the standard of care in the UK) and no follow-up (due to double anonymity). It is worth noting that the aim of this study was to demonstrate the potential capability of our general method and the new paradigm, rather than introduce a new clinical test for PCa that would require larger specialised studies in different settings. The exact mechanism of concordance between leukocyte 3D chromosome conformations and those in primary tumours remains unclear. We hypothesise that it is mediated by the horizontal transfer of epigenetic information, likely through non-coding RNAs, however, dedicated studies are required to investigate this further.

## Conclusions

In conclusion, we have identified subsets of chromosomal conformations in patients’ PBMCs that are strongly indicative of PCa presence and prognosis. These signatures have significant potential for the development of quick diagnostic and prognostic blood tests for PCa and significantly exceed the specificity of the currently used PSA test.

## Supplementary Information

**Additional file 1: Table S1.** Clinical characteristics of the prostate cancer patients participated in the study. **Table S2**. Prostate cancer risk group categories. **Table S3.** List of 425 prostate cancer-related genomic loci tested in the initial array. **Table S4.** Comparison of pathology and EpiSwitch™ results. **Table S5.** Markers for prognostic array stratifications category 1 vs 3 and category 2 vs 3. Top 181 markers produced from the prognostic array. **Table S6.** Markers for high-risk category 3 vs low-risk category 1 and for high-risk category 3 vs intermediate-risk category 2. **Table S7**. Comparison of pathology and EpiSwitch™ results for category 3 vs 1 classifier. **Table S8.** Comparison of pathology and EpiSwitch™ results for category 3 vs 2 classifier.

## Data Availability

The datasets used and/or analysed during the current study are available from the corresponding author on reasonable request.

## Abbreviations

3C: Chromosome conformation capture
ACAT1: Acetyl-CoA acetyltransferase 1
APAF1: Apoptotic peptidase activating factor 1
BMP6: Morphogenetic protein 6
CASP2: Caspase 2
DAPK1: Death-associated protein kinase 1
ERG: ETS transcription factor ERG
ETS1: ETS proto-oncogene 1, transcription factor
GWAS: Genome-wide association studies
HSD3B2: Hydroxy-delta-5-steroid dehydrogenase, 3 beta- and steroid delta-isomerase 2
MAP3K14: Mitogen-activated protein kinase kinase kinase 14
MRI: Magnetic resonance imaging
MSR1: Macrophage scavenger receptor 1
MUC1: Mucin 1
NF-kβ: Nuclear factor-kappa-beta
NIK: Nuclear factor-kappa-beta-inducing kinase
NCCN: National Comprehensive Cancer Network
PBMCs: Peripheral blood mononuclear cells
PCa: Prostate cancer
PCR: Polymerase chain reactions
PSA: Prostate specific antigen
qPCR: Quantitative PCR
SLC22A3: Solute carrier family 22 member 3
VEGFC: Vascular endothelial growth factor C
