# Supplementary material for: Chromatin conformation changes in peripheral blood can detect prostate cancer and stratify disease risk groups
Source: J Transl Med. 2021 Jan 28;19:46. doi: 10.1186/s12967-021-02710-y (PMC7845038; doi:10.1186/s12967-021-02710-y)
Supplement: Supplementary file 1 — Additional file 1: Table S1. Clinical characteristics of the prostate cancer patients participated in the study. Table S2. Prostate cancer risk group categories. Table S3. List of 425 prostate cancer-related genomic loci tested in the initial array. Table S4. Comparison of pathology and EpiSwitch™ results. Table S5. Markers for prognostic array stratifications category 1 vs 3 and category 2 vs 3. Top 181 markers produced from the prognostic array. Table S6. Markers for high-risk category 3 vs low-risk category 1 and for high-risk category 3 vs intermediate-risk category 2. Table S7. Comparison of pathology and EpiSwitch™ results for category 3 vs 1 classifier. Table S8. Comparison of pathology and EpiSwitch™ results for category 3 vs 2 classifier. [file 12967_2021_2710_MOESM1_ESM.docx]

| Table S1. Clinical characteristics of the prostate cancer patients participated in the study. | | | |
| --- | --- | --- | --- |
| **Characteristic** | **Category** | **Number of patients** |  |
| **Gleason score** | ≤6 | 39 |  |
|  | 7 | 54 |  |
|  | 8-10 | 29 |  |
|  | Unknown | 18 |  |
|  | Median | 7 |  |
| **Stage** | 1 | 36 |  |
|  | 2 | 49 |  |
|  | 3 | 25 |  |
|  | 4 | 14 |  |
|  | Unknown | 16 |  |
| **Age** | 45-54 | 12 |  |
|  | 55-64 | 21 |  |
|  | 65-74 | 44 |  |
|  | 75+ | 63 |  |
|  | Unknown | 0 |  |
| **PSA** | <10 | 55 |  |
|  | 10-20 | 23 |  |
|  | >20 | 51 |  |
|  | Unknown | 11 |  |
|  | Median | 12.2 |  |
| **Metastatic patients** | | 21 |  |
| Abbreviation. PSA: prostate specific antigen. | | |  |

| Table S2. Prostate cancer risk group categories. | | | | |
| --- | --- | --- | --- | --- |
| **Category** | **Risk** | **PSA (ng/ml)** | **Gleason score** | **cT stage** |
| 1 | Low | < 10 | ≤6 | T1 - T2a |
| 2 | Intermediate | 10-20 | 7 | T2b |
| 3 | High | >20 | 8-10 | T2c^*^, T3 or T4 |
| ^*^ According to National Comprehensive Cancer Network (NCCN) guidelines 2018 update T2c is considered intermediate risk.  Abbreviations. PSA: prostate specific antigen. | | | | |

Table S3. List of 425 prostate cancer-related genomic loci tested in the initial array.

| **Gene name** | **Array probe count** | **Gene name** | **Array probe count** | **Gene name** | **Array probe count** | **Gene name** | **Array probe count** |
| --- | --- | --- | --- | --- | --- | --- | --- |
| ABHD3 | 20 | CALR | 15 | CREBBP | 20 | EPS15 | 111 |
| ABR | 20 | CAP1 | 20 | CSE1L | 20 | ERBB2 | 20 |
| ACAT1 | 20 | CARS | 20 | CSF2 | 11 | ERBB3 | 20 |
| ACPP | 20 | CASP2 | 20 | CSF2RA | 3 | ERBB4 | 200 |
| ACTA1 | 20 | CASP3 | 20 | CSNK1A1 | 20 | ERG | 67 |
| ACTR2 | 20 | CASP9 | 20 | CTBP1 | 20 | ERRFI1 | 20 |
| ACTR3 | 20 | CAV1 | 20 | CTNNA1 | 43 | ESR1 | 200 |
| ADAM9 | 20 | CBL | 20 | CTNNB1 | 45 | ESR2 | 20 |
| ADRB2 | 20 | CCDC67 | 31 | CTNND1 | 20 | ETS1 | 105 |
| AGAP2 | 15 | CCND1 | 20 | CXCL16 | 19 | ETV1 | 47 |
| AIP | 9 | CCND2 | 20 | CXCR1 | 20 | ETV4 | 20 |
| AKT1 | 20 | CCNE1 | 20 | CXCR2 | 19 | ETV5 | 20 |
| AKT2 | 20 | CCNJ | 6 | CXCR4 | 20 | EZH2 | 31 |
| AKT3 | 122 | CD244 | 20 | CXCR6 | 19 | FASN | 20 |
| AMACR | 20 | CD4 | 20 | CYCS | 18 | FGD4 | 52 |
| AP2M1 | 20 | CD44 | 75 | CYP17A1 | 20 | FGF19 | 20 |
| APAF1 | 26 | CD82 | 20 | CYP19A1 | 149 | FGF2 | 39 |
| APC | 37 | CD8A | 20 | CYP1B1 | 19 | FGF6 | 20 |
| AR | 115 | CDC25A | 20 | DAND5 | 20 | FGF8 | 10 |
| ARAF | 20 | CDC25B | 20 | DAPK1 | 56 | FGFR1 | 20 |
| ARNT | 20 | CDC25C | 20 | DDIT4 | 16 | FGFR4 | 12 |
| ARTN | 20 | CDC37 | 20 | DOK4 | 20 | FHL2 | 20 |
| ASAH1 | 20 | CDC45 | 20 | DPP4 | 103 | FLNA | 9 |
| ATM | 54 | CDH1 | 20 | E2F1 | 20 | FLT1 | 97 |
| AXIN1 | 20 | CDK2 | 2 | E2F4 | 20 | FLT4 | 10 |
| AXL | 20 | CDK4 | 20 | EDN1 | 20 | FN1 | 20 |
| BAD | 20 | CDK6 | 111 | EDNRA | 35 | FOLH1 | 35 |
| BCAR1 | 28 | CDKN1A | 20 | EED | 20 | FOSB | 11 |
| BCL2L1 | 20 | CDKN1B | 20 | EGF | 26 | FOXA1 | 18 |
| BCORL1 | 20 | CDKN2A | 20 | EGFR | 200 | FOXO1 | 21 |
| BGLAP | 11 | CDKN2B | 16 | EIF4E | 20 | FOXO3 | 139 |
| BIRC5 | 4 | CDKN2C | 10 | EIF4EBP1 | 20 | FOXP1 | 200 |
| BMI1 | 20 | CDKN2D | 20 | EIF6 | 20 | FZD1 | 18 |
| BMP6 | 71 | CENPBD1 | 7 | ELAC2 | 20 | GAB1 | 107 |
| BMP7 | 20 | CHAMP1 | 20 | ENPP2 | 74 | GAS6 | 20 |
| BRAF | 20 | CHEK2 | 20 | EP300 | 20 | GLIPR1 | 15 |
| BRCA1 | 20 | CHUK | 29 | EPHA2 | 20 | GNRH1 | 20 |
| BRCA2 | 20 | CLU | 28 | EPHB4 | 20 | GNRHR | 3 |
| CA1 | 20 | COMMD3-BMI1 | 20 | EPHB6 | 6 | GRB2 | 20 |

| **Gene name** | **Array probe count** | **Gene name** | **Array probe count** | **Gene name** | **Array probe count** | **Gene name** | **Array probe count** |
| --- | --- | --- | --- | --- | --- | --- | --- |
| GSK3B | 92 | KLK4 | 17 | MIR34A | 20 | NFKB1 | 66 |
| GSTP1 | 20 | KRAS | 20 | MIR361 | 20 | NFKBIA | 20 |
| HDAC1 | 20 | LPAR1 | 30 | MIR365A | 5 | NGF | 21 |
| HDAC3 | 20 | LPAR2 | 5 | MIR376A1 | 9 | NKX3-1 | 20 |
| HGF | 27 | LPAR3 | 31 | MIR454 | 11 | NOVA1 | 34 |
| HIF1A | 20 | LPAR4 | 5 | MIR500A | 20 | NOX5 | 43 |
| HIPK2 | 20 | LRP5 | 20 | MIR582 | 3 | NPDC1 | 1 |
| HRAS | 6 | LRP6 | 35 | MIR619 | 20 | NR0B1 | 10 |
| HSD3B1 | 20 | MAGEA11 | 20 | MIR636 | 20 | NR3C1 | 25 |
| HSD3B2 | 20 | MAP2K1 | 20 | MIR648 | 20 | NR4A3 | 20 |
| HSP90AA1 | 20 | MAP2K2 | 20 | MIR671 | 20 | NRAS | 20 |
| HSP90AB1 | 20 | MAP2K5 | 196 | MIR766 | 15 | NRIP1 | 69 |
| HSPA1A | 20 | MAP3K14 | 20 | MIR877 | 13 | NTF3 | 31 |
| HSPB1 | 20 | MAP3K2 | 24 | MIR887 | 18 | NTRK1 | 20 |
| IGF1 | 20 | MAPK1 | 20 | MIR93 | 20 | PA2G4 | 20 |
| IGF1R | 102 | MAPK3 | 20 | MIR98 | 17 | PAQR7 | 20 |
| IGFBP3 | 14 | MAPKAP1 | 31 | MIRLET7G | 3 | PAX8 | 20 |
| IGFBP5 | 20 | MCM7 | 20 | MLST8 | 2 | PCBP2 | 20 |
| IL16 | 82 | MDM2 | 20 | MMP14 | 20 | PCYT1A | 20 |
| IL2 | 2 | MDM4 | 23 | MMP9 | 20 | PDGFA | 20 |
| IL6 | 20 | MED1 | 20 | MSMB | 20 | PDGFB | 20 |
| IL6R | 20 | MEN1 | 20 | MSR1 | 200 | PDGFRA | 24 |
| IL8 | 17 | MET | 105 | MTA1 | 1 | PDGFRB | 20 |
| INPPL1 | 20 | MIF | 12 | MTCH1 | 20 | PDPK1 | 20 |
| INS | 17 | MIR125B1 | 4 | MTOR | 32 | PIAS1 | 89 |
| IRAK1 | 20 | MIR149 | 3 | MTRR | 20 | PIAS2 | 21 |
| IRS1 | 20 | MIR151A | 31 | MUC1 | 7 | PIAS3 | 20 |
| ITK | 24 | MIR152 | 15 | MYB | 20 | PIAS4 | 20 |
| JAK1 | 20 | MIR16-1 | 20 | MYC | 20 | PIK3C2A | 20 |
| JAK2 | 20 | MIR183 | 20 | NCOA1 | 170 | PIK3C2B | 21 |
| JAK3 | 20 | MIR197 | 7 | NCOA2 | 200 | PIK3C2G | 200 |
| JUN | 20 | MIR204 | 20 | NCOA3 | 20 | PIK3CA | 20 |
| KAT7 | 20 | MIR222 | 6 | NCOA4 | 18 | PIK3CB | 21 |
| KCNH2 | 20 | MIR224 | 20 | NCOR1 | 35 | PIK3CD | 20 |
| KDM6B | 20 | MIR23B | 20 | NCOR2 | 26 | PIK3CG | 20 |
| KIT | 107 | MIR24-2 | 20 | NEDD4 | 85 | PIK3R1 | 81 |
| KLF4 | 20 | MIR26B | 3 | NEDD4L | 128 | PIK3R2 | 20 |
| KLK2 | 20 | MIR27B | 20 | NET1 | 20 | PLD1 | 189 |
| KLK3 | 8 | MIR335 | 20 | NF1 | 190 | PLD2 | 20 |
| **Gene name** | **Array probe count** | **Gene name** | **Array probe count** | **Gene name** | **Array probe count** | **Gene name** | **Array probe count** |
| PLD3 | 20 | PXN | 20 | SFTPA1 | 20 | TGFB1I1 | 20 |
| PML | 20 | RAB9B | 16 | SFTPA2 | 8 | TGFB2 | 75 |
| POU2F1 | 26 | RAD51 | 9 | SHC1 | 11 | TGFB3 | 15 |
| POU2F2 | 20 | RAF1 | 20 | SIRT1 | 20 | TGFBR1 | 20 |
| PPP1CA | 20 | RAN | 20 | SKP2 | 16 | TGFBR2 | 65 |
| PPP2CA | 20 | RB1 | 82 | SLC22A3 | 56 | TIMP1 | 20 |
| PRKCA | 148 | RCHY1 | 20 | SMAD3 | 20 | TMF1 | 20 |
| PRKCB | 176 | REL | 25 | SMAD4 | 67 | TMPRSS2 | 36 |
| PRKCD | 20 | RGS6 | 200 | SMARCE1 | 20 | TNK2 | 20 |
| PRKCG | 20 | RHEB | 20 | SOAT1 | 20 | TOP2A | 20 |
| PRKCH | 200 | RHOA | 20 | SOS1 | 103 | TOP2B | 24 |
| PRKCI | 43 | RICTOR | 38 | SOX9 | 20 | TP53 | 15 |
| PRKCQ | 59 | RNASEL | 13 | SP1 | 20 | TRAF3 | 20 |
| PRKCZ | 20 | RNF14 | 20 | SPDEF | 17 | TRAF6 | 20 |
| PRSS3 | 23 | RNF20 | 20 | SPINK1 | 20 | TSC1 | 20 |
| PRSS8 | 20 | RNF40 | 20 | SPOPL | 20 | TSC2 | 2 |
| PSAP | 20 | ROCK1 | 67 | SRC | 20 | TUBB | 4 |
| PSCA | 20 | ROR2 | 193 | SRD5A1 | 13 | VEGFA | 20 |
| PSG1 | 19 | RPS6KA1 | 20 | SRD5A2 | 79 | VEGFC | 57 |
| PTEN | 23 | RPS6KB1 | 20 | SRD5A3 | 20 | VIM | 9 |
| PTGS2 | 20 | RPTOR | 68 | SREBF1 | 20 | WAS | 12 |
| PTK2B | 54 | RREB1 | 85 | SRY | 3 | WNT1 | 20 |
| PTK7 | 20 | RYBP | 20 | STAT3 | 20 | WNT2 | 40 |
| PTPN11 | 20 | S100A4 | 20 | SUZ12 | 20 | WNT3 | 20 |
| PTPN12 | 20 | S100P | 20 | SVIL | 51 | WNT5A | 23 |
| PTPN14 | 135 | SAGE1 | 20 | TBC1D8 | 43 | ZAP70 | 20 |
| PTPRF | 20 | SATB1 | 20 | TERT | 20 | ZFAND1 | 20 |
| PTPRR | 200 | SCGB1A1 | 20 | TGFB1 | 20 | ZMYND10 | 20 |
| PTPRT | 200 |  |  |  |  | **Total** | **14241** |

| Table S4.  Comparison of pathology and EpiSwitch™ results. | | |
| --- | --- | --- |
|  | Pathology results | |
| EpiSwitch™ diagnosis | PCa | Healthy |
| PCa | 8 | 2 |
| Healthy | 2 | 8 |

| Results from classification of blinded samples (n=20). | | |
| --- | --- | --- |
| **Statistic** | **Value** | **95% CI** |
| Sensitivity | 80.00% | 44.39% to 97.48% |
| Specificity | 80.00% | 44.39% to 97.48% |
| Positive Likelihood Ratio | 4.00 | 1.11 to 14.35 |
| Negative Likelihood Ratio | 0.25 | 0.07 to 0.90 |
| Disease prevalence (*) | 50.00% | 27.20% to 72.80% |
| Positive Predictive Value (*) | 80.00% | 52.71% to 93.49% |
| Negative Predictive Value (*) | 80.00% | 52.71% to 93.49% |
| (*) These values are dependent on disease prevalence.  Abbreviations. 95% CI: 95% confidence interval. | | |

| Table S5. Markers for prognostic array stratifications category 1 vs 3 and category 2 vs 3. Top 181 markers produced from the prognostic array. | | | | | | | | | | |
| --- | --- | --- | --- | --- | --- | --- | --- | --- | --- | --- |
| **Probes** | **GeneLocus** | **logFC** | **AveExpr** | **t** | **P.Value** | **adj.P.Val** | **B** | **FC** | **FC_1** | **Binary** |
| ACAT1_11_107955219_107960166_108013361_108018367_FF | ACAT1 | -0.436725529 | -0.436725529 | -11.90723067 | 1.37E-07 | 3.10E-05 | 8.114204006 | 0.738809576 | -1.353528748 | -1 |
| ACTA1_1_229547333_229551721_229600994_229605798_FR | ACTA1 | 0.417850291 | 0.417850291 | 8.736657363 | 2.98E-06 | 0.000260244 | 5.025483662 | 1.335935441 | 1.335935441 | 1 |
| AKT3_1_243680126_243690814_243946602_243948601_FR | AKT3 | 0.652970743 | 0.652970743 | 16.8960264 | 3.63E-09 | 3.08E-06 | 11.560433 | 1.572402697 | 1.572402697 | 1 |
| AKT3_1_243680126_243690814_243915703_243918596_FR | AKT3 | 0.598435451 | 0.598435451 | 11.73324265 | 1.59E-07 | 3.45E-05 | 7.966660757 | 1.514073719 | 1.514073719 | 1 |
| AKT3_1_243680126_243690814_243727939_243733240_FF | AKT3 | 0.520843747 | 0.520843747 | 19.9294303 | 6.33E-10 | 1.07E-06 | 13.10148999 | 1.434794129 | 1.434794129 | 1 |
| AKT3_1_243680126_243690814_243860421_243862288_FR | AKT3 | 0.410316196 | 0.410316196 | 12.44251976 | 8.76E-08 | 2.55E-05 | 8.554504322 | 1.328977055 | 1.328977055 | 1 |
| APAF1_12_99061113_99062942_99098781_99108240_FF | APAF1 | -0.441488336 | -0.441488336 | -13.23940926 | 4.63E-08 | 1.71E-05 | 9.174110234 | 0.736374546 | -1.358004571 | -1 |
| APC_5_112020873_112029146_112079758_112082452_FF | APC | 0.399930381 | 0.399930381 | 6.922678201 | 2.63E-05 | 0.000993557 | 2.789251424 | 1.319444238 | 1.319444238 | 1 |
| AR_X_66792540_66795953_66818342_66825862_RF | AR | -0.33854166 | -0.33854166 | -6.221155823 | 6.77E-05 | 0.001660581 | 1.810263084 | 0.790840324 | -1.264477758 | -1 |
| AR_X_66736338_66750729_66875649_66881776_RR | AR | 0.760948793 | 0.760948793 | 21.04618466 | 3.54E-10 | 7.78E-07 | 13.59167252 | 1.694604721 | 1.694604721 | 1 |
| AR_X_66736338_66750729_66906874_66911452_RR | AR | 0.563742659 | 0.563742659 | 20.89428568 | 3.82E-10 | 7.78E-07 | 13.52715233 | 1.478098751 | 1.478098751 | 1 |
| AR_X_66750729_66754087_66950367_66956132_FR | AR | 0.528294859 | 0.528294859 | 9.248783162 | 1.71E-06 | 0.000187011 | 5.588100983 | 1.442223604 | 1.442223604 | 1 |
| AR_X_66818342_66825862_66950367_66956132_FF | AR | 0.388697407 | 0.388697407 | 10.14296783 | 6.90E-07 | 9.11E-05 | 6.507167389 | 1.309210798 | 1.309210798 | 1 |
| AR_X_66911452_66916150_66950367_66956132_RR | AR | 0.377269056 | 0.377269056 | 7.449040178 | 1.34E-05 | 0.000639022 | 3.480123787 | 1.298880815 | 1.298880815 | 1 |
| AR_X_66736338_66750729_66875649_66881776_FR | AR | 0.370736884 | 0.370736884 | 5.172651841 | 0.000316014 | 0.004216579 | 0.216960602 | 1.293013093 | 1.293013093 | 1 |
| ATM_11_108112750_108115594_108208085_108223747_FR | ATM | -0.370811815 | -0.370811815 | -8.707610279 | 3.07E-06 | 0.000260244 | 4.992731905 | 0.773347206 | -1.293080251 | -1 |
| ATM_11_108155279_108156687_108208085_108223747_RR | ATM | 0.363186489 | 0.363186489 | 6.602286154 | 4.02E-05 | 0.001238401 | 2.350592936 | 1.28626374 | 1.28626374 | 1 |
| BMP6_6_7724582_7733496_7801590_7806316_FF | BMP6 | -0.468602239 | -0.468602239 | -8.973309325 | 2.30E-06 | 0.000229279 | 5.288915333 | 0.722664415 | -1.383768149 | -1 |
| BMP6_6_7724582_7733496_7743581_7746369_FR | BMP6 | -0.388036314 | -0.388036314 | -5.889142945 | 0.000108497 | 0.002198909 | 1.322801574 | 0.764169025 | -1.30861101 | -1 |
| CD44_11_35172600_35178637_35204720_35210484_FR | CD44 | -0.398048925 | -0.398048925 | -5.668665177 | 0.000149604 | 0.002652749 | 0.990360448 | 0.758883891 | -1.317724638 | -1 |
| CDH1_16_68794947_68799115_68857468_68863222_FR | CDH1 | 0.49540487 | 0.49540487 | 10.65592887 | 4.22E-07 | 6.84E-05 | 7.000965624 | 1.409716315 | 1.409716315 | 1 |
| CTNNB1_3_41228301_41234483_41281934_41304993_FR | CTNNB1 | 0.427937487 | 0.427937487 | 10.8349435 | 3.57E-07 | 6.15E-05 | 7.167935447 | 1.345308914 | 1.345308914 | 1 |
| DPP4_2_162933505_162942299_162961246_162964936_FR | DPP4 | -0.512949291 | -0.512949291 | -14.57522664 | 1.71E-08 | 1.09E-05 | 10.1259903 | 0.700788356 | -1.426964348 | -1 |
| DPP4_2_162946178_162949954_162972154_162979139_RF | DPP4 | -0.445665864 | -0.445665864 | -3.573437421 | 0.004427816 | 0.023189841 | -2.491256189 | 0.734245353 | -1.361942565 | -1 |
| EGFR_7_55080257_55086091_55224588_55235839_RR | EGFR | -0.493404965 | -0.493404965 | -11.0577665 | 2.91E-07 | 5.37E-05 | 7.372042198 | 0.710346599 | -1.407763479 | -1 |
| EPS15_1_51804255_51813510_51945067_51946855_FF | EPS15 | 0.386206462 | 0.386206462 | 8.698890643 | 3.10E-06 | 0.000260244 | 4.982882133 | 1.306952276 | 1.306952276 | 1 |
| ERBB4_2_213060845_213063716_213336205_213346911_FR | ERBB4 | -0.681305564 | -0.681305564 | -14.78217408 | 1.48E-08 | 1.00E-05 | 10.26456915 | 0.623600693 | -1.603590265 | -1 |
| ERBB4_2_212789287_212798405_212962659_212969505_FR | ERBB4 | -0.435496836 | -0.435496836 | -5.826671395 | 0.000118755 | 0.002319347 | 1.229315586 | 0.739439062 | -1.352376485 | -1 |
| ERBB4_2_213052672_213059531_213336205_213346911_FR | ERBB4 | -0.425728775 | -0.425728775 | -12.76218491 | 6.75E-08 | 2.15E-05 | 8.808033687 | 0.744462573 | -1.343250872 | -1 |
| ERBB4_2_212789287_212798405_212846041_212850086_RF | ERBB4 | -0.356744537 | -0.356744537 | -6.538422232 | 4.38E-05 | 0.001298756 | 2.261469357 | 0.780924761 | -1.280533093 | -1 |
| ERBB4_2_212556994_212565232_212622803_212628844_FR | ERBB4 | 0.688270832 | 0.688270832 | 10.79268571 | 3.71E-07 | 6.29E-05 | 7.128763987 | 1.611351047 | 1.611351047 | 1 |
| ERBB4_2_213182054_213190315_213317793_213323368_RR | ERBB4 | 0.447473513 | 0.447473513 | 10.33352546 | 5.73E-07 | 8.45E-05 | 6.693320715 | 1.363650103 | 1.363650103 | 1 |
| ERBB4_2_212622803_212628844_212789287_212798405_RF | ERBB4 | 0.416082272 | 0.416082272 | 5.631318317 | 0.000158077 | 0.002758619 | 0.933355245 | 1.334299258 | 1.334299258 | 1 |
| ERBB4_2_213151813_213159540_213182054_213190315_FF | ERBB4 | 0.378853461 | 0.378853461 | 3.217386837 | 0.008284402 | 0.035699071 | -3.122932982 | 1.300308063 | 1.300308063 | 1 |
| ERBB4_2_212556994_212565232_212858137_212868453_FF | ERBB4 | 0.359671724 | 0.359671724 | 6.923758849 | 2.62E-05 | 0.000993557 | 2.790707369 | 1.283133896 | 1.283133896 | 1 |
| ERG_21_39895678_39899145_39984806_39991905_RF | ERG | -0.425291613 | -0.425291613 | -11.67074071 | 1.68E-07 | 3.57E-05 | 7.913111034 | 0.744688192 | -1.342843905 | -1 |
| ESR1_6_152151654_152158599_152307023_152319013_RF | ESR1 | -0.334294612 | -0.334294612 | -9.744626114 | 1.03E-06 | 0.000124223 | 6.107253454 | 0.793171853 | -1.260760825 | -1 |
| ETS1_11_128387417_128389914_128489818_128498866_FF | ETS1 | -0.529524556 | -0.529524556 | -8.680536392 | 3.17E-06 | 0.000260244 | 4.962121749 | 0.692783005 | -1.443453423 | -1 |
| ETS1_11_128431527_128436474_128489818_128498866_RF | ETS1 | -0.370215655 | -0.370215655 | -5.977392038 | 9.56E-05 | 0.002017625 | 1.453907443 | 0.77366684 | -1.292546027 | -1 |
| ETS1_11_128342943_128345136_128399358_128409879_FF | ETS1 | -0.344088814 | -0.344088814 | -4.169479623 | 0.001593588 | 0.012099379 | -1.449395395 | 0.787805386 | -1.269349026 | -1 |
| ETS1_11_128342943_128345136_128489818_128498866_FF | ETS1 | -0.342780154 | -0.342780154 | -4.97505672 | 0.000429841 | 0.005055727 | -0.100832117 | 0.788520324 | -1.26819813 | -1 |
| ETV1_7_13928482_13938998_14075713_14080964_FR | ETV1 | -0.438646448 | -0.438646448 | -12.61661413 | 7.60E-08 | 2.29E-05 | 8.693428053 | 0.737826521 | -1.355332144 | -1 |
| ETV1_7_13928482_13938998_14040827_14042620_FR | ETV1 | -0.420116215 | -0.420116215 | -7.525930383 | 1.22E-05 | 0.000603601 | 3.57803973 | 0.747364419 | -1.338035334 | -1 |
| FOLH1_11_49157869_49163274_49234427_49241370_FF | FOLH1 | -0.327026922 | -0.327026922 | -4.300654557 | 0.001279787 | 0.010350202 | -1.224472556 | 0.7971776 | -1.254425613 | -1 |
| FOLH1_11_49214976_49217503_49234427_49241370_RF | FOLH1 | 0.418767287 | 0.418767287 | 6.637609483 | 3.83E-05 | 0.00120659 | 2.399644945 | 1.336784849 | 1.336784849 | 1 |
| FOLH1_11_49157869_49163274_49193665_49198286_RR | FOLH1 | 0.359712744 | 0.359712744 | 10.54139329 | 4.70E-07 | 7.47E-05 | 6.892707121 | 1.28317038 | 1.28317038 | 1 |
| GLIPR1_12_75847260_75849629_75907812_75913956_FR | GLIPR1 | -0.345396052 | -0.345396052 | -5.155710032 | 0.000324389 | 0.004293149 | 0.189926553 | 0.787091873 | -1.270499715 | -1 |
| GSK3B_3_119542459_119548768_119722182_119724690_FR | GSK3B | 0.449273935 | 0.449273935 | 10.95895529 | 3.18E-07 | 5.68E-05 | 7.282033839 | 1.365352943 | 1.365352943 | 1 |
| HGF_7_81320024_81325883_81430055_81434910_FF | HGF | -0.43281007 | -0.43281007 | -4.979466125 | 0.000426875 | 0.00504444 | -0.093681464 | 0.740817421 | -1.349860265 | -1 |
| IGFBP5_2_217560127_217567417_217584428_217589578_FR | IGFBP5 | 0.375003854 | 0.375003854 | 12.14650233 | 1.12E-07 | 2.78E-05 | 8.313515985 | 1.296843019 | 1.296843019 | 1 |
| IL16_15_81429756_81433873_81539851_81547011_FR | IL16 | -0.481896248 | -0.481896248 | -9.127530593 | 1.95E-06 | 0.000208593 | 5.457383883 | 0.716035863 | -1.396578093 | -1 |
| IL6_7_22721376_22727129_22765455_22766829_FR | IL6 | 0.364631786 | 0.364631786 | 4.801938715 | 0.00056537 | 0.005957969 | -0.383664127 | 1.28755297 | 1.28755297 | 1 |
| JUN_1_59244918_59246918_59258836_59260597_RF | JUN | -0.318607525 | -0.318607525 | -5.099115371 | 0.000354116 | 0.004543222 | 0.099326736 | 0.801843436 | -1.247126254 | -1 |
| KIT_4_55553401_55555465_55610649_55618756_FR | KIT | 0.392634635 | 0.392634635 | 7.232977499 | 1.76E-05 | 0.000779657 | 3.200921573 | 1.312788617 | 1.312788617 | 1 |
| KRAS_12_25363357_25368892_25413300_25418096_FR | KRAS | 0.414148209 | 0.414148209 | 11.99343016 | 1.28E-07 | 3.02E-05 | 8.186481963 | 1.332511707 | 1.332511707 | 1 |
| LPAR3_1_85307233_85315383_85371057_85373099_RR | LPAR3 | -0.38895037 | -0.38895037 | -13.49428502 | 3.80E-08 | 1.61E-05 | 9.363780155 | 0.76368502 | -1.309440376 | -1 |
| LPAR3_1_85265679_85268722_85307233_85315383_RR | LPAR3 | 0.402766702 | 0.402766702 | 11.08898448 | 2.82E-07 | 5.32E-05 | 7.400314167 | 1.322040801 | 1.322040801 | 1 |
| MAP2K5_15_67818519_67824995_68067482_68072379_FR | MAP2K5 | 0.404437029 | 0.404437029 | 9.01303797 | 2.20E-06 | 0.000221829 | 5.332553143 | 1.323572323 | 1.323572323 | 1 |
| MAPKAP1_9_128370452_128376700_128393518_128397379_RF | MAPKAP1 | -0.396294954 | -0.396294954 | -12.18592711 | 1.08E-07 | 2.78E-05 | 8.345964775 | 0.759807072 | -1.316123575 | -1 |
| MIR454_17_57199107_57202160_57227315_57228782_FF | MIR454 | 0.373313547 | 0.373313547 | 4.540644288 | 0.000861835 | 0.007908614 | -0.818047279 | 1.295324487 | 1.295324487 | 1 |
| MIR98_X_53595032_53600487_53628991_53630033_RR | MIR98 | 0.742460493 | 0.742460493 | 23.55441603 | 1.06E-10 | 5.41E-07 | 14.56868412 | 1.673026728 | 1.673026728 | 1 |
| MIR98_X_53608013_53611637_53628991_53630033_RR | MIR98 | 0.67511652 | 0.67511652 | 13.76185645 | 3.10E-08 | 1.43E-05 | 9.558686586 | 1.596725728 | 1.596725728 | 1 |
| MSR1_8_16213140_16220021_16405541_16412741_RR | MSR1 | -0.589823054 | -0.589823054 | -8.317534796 | 4.77E-06 | 0.000337002 | 4.54380866 | 0.664424394 | -1.50506214 | -1 |
| MSR1_8_16195878_16203315_16396849_16400398_FF | MSR1 | -0.419369028 | -0.419369028 | -3.516010141 | 0.004895359 | 0.024952597 | -2.59289798 | 0.747751587 | -1.337342532 | -1 |
| MSR1_8_16045879_16049928_16079226_16088483_FF | MSR1 | -0.385893508 | -0.385893508 | -10.14385541 | 6.89E-07 | 9.11E-05 | 6.508042059 | 0.765304873 | -1.306668799 | -1 |
| MSR1_8_16142611_16149459_16195878_16203315_FF | MSR1 | -0.337812738 | -0.337812738 | -6.009884545 | 9.13E-05 | 0.00198016 | 1.501898146 | 0.791239998 | -1.263839041 | -1 |
| MSR1_8_16142611_16149459_16396849_16400398_RF | MSR1 | -0.328903769 | -0.328903769 | -5.188518336 | 0.000308377 | 0.004162163 | 0.242242178 | 0.796141201 | -1.256058596 | -1 |
| MSR1_8_16251114_16260512_16462527_16467449_FF | MSR1 | -0.318902812 | -0.318902812 | -7.84658429 | 8.28E-06 | 0.000473286 | 3.978390344 | 0.801679333 | -1.247381538 | -1 |
| MSR1_8_16195878_16203315_16433596_16442100_FR | MSR1 | 0.420145921 | 0.420145921 | 3.761840375 | 0.003192302 | 0.018914227 | -2.159017761 | 1.338062886 | 1.338062886 | 1 |
| NCOA1_2_24829718_24833469_24853776_24866328_RR | NCOA1 | 0.383599799 | 0.383599799 | 7.061388057 | 2.19E-05 | 0.000893094 | 2.974854782 | 1.304593006 | 1.304593006 | 1 |
| NCOA1_2_24696090_24698819_24840193_24848780_RF | NCOA1 | 0.376458226 | 0.376458226 | 8.007884338 | 6.84E-06 | 0.000434693 | 4.175013545 | 1.298151018 | 1.298151018 | 1 |
| NCOA1_2_24672976_24676297_24840193_24848780_RF | NCOA1 | 0.368303096 | 0.368303096 | 8.47574282 | 3.98E-06 | 0.000302485 | 4.72794807 | 1.290833654 | 1.290833654 | 1 |
| NEDD4L_18_55713082_55720762_55811019_55814883_RR | NEDD4L | -0.444311776 | -0.444311776 | -12.22570821 | 1.05E-07 | 2.78E-05 | 8.378595915 | 0.734934827 | -1.360664869 | -1 |
| NEDD4L_18_55713082_55720762_55848311_55850861_RR | NEDD4L | -0.390337066 | -0.390337066 | -12.14497251 | 1.12E-07 | 2.78E-05 | 8.312254649 | 0.76295133 | -1.310699595 | -1 |
| NEDD4L_18_55713082_55720762_55882560_55885168_RR | NEDD4L | -0.37571287 | -0.37571287 | -12.60684517 | 7.66E-08 | 2.29E-05 | 8.685686665 | 0.770724485 | -1.297480512 | -1 |
| NEDD4L_18_55713082_55720762_55869254_55872326_RR | NEDD4L | -0.360925867 | -0.360925867 | -11.91420916 | 1.36E-07 | 3.10E-05 | 8.120075504 | 0.778664702 | -1.284249816 | -1 |
| NEDD4L_18_55713082_55720762_55774243_55779941_RR | NEDD4L | -0.35657074 | -0.35657074 | -9.240361998 | 1.73E-06 | 0.000187011 | 5.579071341 | 0.781018843 | -1.28037884 | -1 |
| NEDD4L_18_55713082_55720762_55961376_55965188_RR | NEDD4L | -0.356380382 | -0.356380382 | -8.945405461 | 2.37E-06 | 0.00023 | 5.258165908 | 0.781121902 | -1.28020991 | -1 |
| NEDD4L_18_55713082_55720762_55784812_55787427_RR | NEDD4L | -0.354821703 | -0.354821703 | -12.0183087 | 1.25E-07 | 3.02E-05 | 8.207242683 | 0.781966277 | -1.278827526 | -1 |
| NEDD4L_18_55713082_55720762_55986600_55989306_RR | NEDD4L | -0.349236239 | -0.349236239 | -11.79725824 | 1.51E-07 | 3.34E-05 | 8.021205936 | 0.784999566 | -1.273886055 | -1 |
| NEDD4L_18_55713082_55720762_55927256_55929658_RR | NEDD4L | -0.343996768 | -0.343996768 | -8.731068109 | 3.00E-06 | 0.000260244 | 5.019188722 | 0.787855651 | -1.269268043 | -1 |
| NEDD4L_18_55713082_55720762_55950636_55953432_RR | NEDD4L | -0.326323266 | -0.326323266 | -12.21462858 | 1.06E-07 | 2.78E-05 | 8.36951882 | 0.797566509 | -1.253813932 | -1 |
| NEDD4L_18_55713082_55720762_55876126_55882560_RR | NEDD4L | -0.319472154 | -0.319472154 | -10.93796227 | 3.24E-07 | 5.69E-05 | 7.262808228 | 0.801363023 | -1.2478739 | -1 |
| NF1_17_29477103_29483764_29709143_29714529_FR | NF1 | 0.640366513 | 0.640366513 | 17.71768057 | 2.20E-09 | 2.49E-06 | 12.01137937 | 1.5587251 | 1.5587251 | 1 |
| NF1_17_29659279_29666456_29709143_29714529_FR | NF1 | 0.558103066 | 0.558103066 | 14.14945638 | 2.33E-08 | 1.29E-05 | 9.83363673 | 1.472332041 | 1.472332041 | 1 |
| NF1_17_29477103_29483764_29651799_29657368_FF | NF1 | 0.451959985 | 0.451959985 | 13.17645951 | 4.86E-08 | 1.71E-05 | 9.126648681 | 1.367897363 | 1.367897363 | 1 |
| NF1_17_29629862_29634257_29659279_29666456_RF | NF1 | 0.36665184 | 0.36665184 | 7.2650224 | 1.69E-05 | 0.000765315 | 3.242712391 | 1.289357057 | 1.289357057 | 1 |
| NFKB1_4_103436488_103442700_103548256_103555520_FR | NFKB1 | 0.79980903 | 0.79980903 | 13.11977478 | 5.08E-08 | 1.72E-05 | 9.083698686 | 1.740870671 | 1.740870671 | 1 |
| NFKB1_4_103425294_103430395_103548256_103555520_RR | NFKB1 | 0.430874236 | 0.430874236 | 14.09753934 | 2.42E-08 | 1.29E-05 | 9.797304101 | 1.348050213 | 1.348050213 | 1 |
| NOVA1_14_26999345_27006013_27046501_27053973_FR | NOVA1 | -0.461947202 | -0.461947202 | -11.22100125 | 2.51E-07 | 5.00E-05 | 7.519006961 | 0.726005709 | -1.377399637 | -1 |
| NOVA1_14_26986332_26987866_27070837_27086602_FF | NOVA1 | -0.325885172 | -0.325885172 | -7.120645256 | 2.03E-05 | 0.000844533 | 3.053363996 | 0.797808737 | -1.253433252 | -1 |
| NR4A3_9_102621891_102624499_102636939_102640160_FR | NR4A3 | -0.326733183 | -0.326733183 | -6.549075716 | 4.32E-05 | 0.001283905 | 2.276375868 | 0.797339926 | -1.254170233 | -1 |
| PIAS2_18_44419921_44425175_44533399_44538938_FF | PIAS2 | -0.376922678 | -0.376922678 | -4.811482513 | 0.000556831 | 0.005918513 | -0.367966827 | 0.770078446 | -1.298569003 | -1 |
| PIK3C2A_11_17158103_17163660_17253125_17255535_FR | PIK3C2A | -0.412653759 | -0.412653759 | -9.581839254 | 1.21E-06 | 0.000138409 | 5.939488583 | 0.751240236 | -1.331132109 | -1 |
| PIK3C2G_12_18503466_18517448_18605599_18615448_FF | PIK3C2G | -0.397193613 | -0.397193613 | -6.065111351 | 8.44E-05 | 0.001886444 | 1.583120211 | 0.759333934 | -1.316943647 | -1 |
| PIK3C2G_12_18682015_18689955_18755082_18765416_FF | PIK3C2G | -0.349259284 | -0.349259284 | -7.872086105 | 8.03E-06 | 0.000473286 | 4.009686303 | 0.784987026 | -1.273906404 | -1 |
| PIK3C2G_12_18503466_18517448_18653437_18654550_FF | PIK3C2G | 0.813156179 | 0.813156179 | 32.76884362 | 3.04E-12 | 3.10E-08 | 17.09863144 | 1.757051136 | 1.757051136 | 1 |
| PIK3C2G_12_18503466_18517448_18800920_18805991_FR | PIK3C2G | 0.52068763 | 0.52068763 | 19.49409228 | 8.00E-10 | 1.16E-06 | 12.9000512 | 1.434638875 | 1.434638875 | 1 |
| PIK3C2G_12_18503466_18517448_18586459_18591749_FR | PIK3C2G | 0.500422551 | 0.500422551 | 22.08623513 | 2.12E-10 | 7.17E-07 | 14.016338 | 1.414627832 | 1.414627832 | 1 |
| PIK3C2G_12_18503466_18517448_18623979_18629934_FR | PIK3C2G | 0.454500507 | 0.454500507 | 14.21973094 | 2.21E-08 | 1.29E-05 | 9.882576362 | 1.370308292 | 1.370308292 | 1 |
| PIK3C2G_12_18466993_18474305_18503466_18517448_RF | PIK3C2G | 0.418315764 | 0.418315764 | 7.846493305 | 8.28E-06 | 0.000473286 | 3.978278546 | 1.336366539 | 1.336366539 | 1 |
| PIK3C2G_12_18407299_18408850_18503466_18517448_RF | PIK3C2G | 0.417537091 | 0.417537091 | 12.19551672 | 1.08E-07 | 2.78E-05 | 8.35384099 | 1.335645449 | 1.335645449 | 1 |
| PIK3C2G_12_18503466_18517448_18748288_18755082_FR | PIK3C2G | 0.412545687 | 0.412545687 | 8.404871486 | 4.32E-06 | 0.000313738 | 4.645813078 | 1.331032398 | 1.331032398 | 1 |
| PIK3C2G_12_18409429_18411730_18662602_18673822_RF | PIK3C2G | 0.404135265 | 0.404135265 | 7.905355237 | 7.72E-06 | 0.000473286 | 4.050395593 | 1.323295505 | 1.323295505 | 1 |
| PIK3C2G_12_18466993_18474305_18765637_18775643_FR | PIK3C2G | 0.401350605 | 0.401350605 | 10.51089877 | 4.83E-07 | 7.57E-05 | 6.863693215 | 1.32074377 | 1.32074377 | 1 |
| PRKCB_16_23929937_23938239_24143206_24145438_FR | PRKCB | 0.359909066 | 0.359909066 | 12.83614979 | 6.36E-08 | 2.09E-05 | 8.865730305 | 1.283345005 | 1.283345005 | 1 |
| PRKCH_14_61911060_61914582_62023126_62035192_FR | PRKCH | -0.411325245 | -0.411325245 | -5.079346062 | 0.000365169 | 0.004598091 | 0.067573492 | 0.751932339 | -1.329906892 | -1 |
| PRKCH_14_61772357_61775932_61963825_61969638_RR | PRKCH | -0.335493699 | -0.335493699 | -7.529186724 | 1.22E-05 | 0.000603601 | 3.582169975 | 0.792512887 | -1.261809134 | -1 |
| PTGS2_1_186630471_186639286_186675090_186678395_RR | PTGS2 | -0.481332802 | -0.481332802 | -6.768676224 | 3.22E-05 | 0.001116912 | 2.580151439 | 0.716315566 | -1.396032765 | -1 |
| PTPN14_1_214555543_214567111_214696754_214699528_RR | PTPN14 | -0.656412698 | -0.656412698 | -6.179949212 | 7.18E-05 | 0.001709697 | 1.750618263 | 0.634453925 | -1.576158584 | -1 |
| PTPN14_1_214555543_214567111_214590581_214592230_FF | PTPN14 | -0.341216493 | -0.341216493 | -6.202805991 | 6.95E-05 | 0.001671497 | 1.783732259 | 0.789375423 | -1.266824341 | -1 |
| PTPN14_1_214512778_214523707_214646434_214652454_FR | PTPN14 | -0.318133487 | -0.318133487 | -4.495773109 | 0.000927417 | 0.008276791 | -0.89351305 | 0.802106947 | -1.246716543 | -1 |
| PTPN14_1_214555543_214567111_214643240_214644608_FR | PTPN14 | 0.743894345 | 0.743894345 | 14.95502699 | 1.31E-08 | 9.50E-06 | 10.37860359 | 1.674690326 | 1.674690326 | 1 |
| PTPRR_12_71045661_71048060_71347632_71356891_FR | PTPRR | -0.321158046 | -0.321158046 | -5.612244267 | 0.0001626 | 0.002808651 | 0.904163728 | 0.80042712 | -1.249332981 | -1 |
| PTPRR_12_71085097_71096639_71123929_71126257_FR | PTPRR | 0.550492009 | 0.550492009 | 13.87391165 | 2.85E-08 | 1.38E-05 | 9.639060097 | 1.464585085 | 1.464585085 | 1 |
| PTPRR_12_71085097_71096639_71150835_71153565_FR | PTPRR | 0.37642789 | 0.37642789 | 7.745377455 | 9.35E-06 | 0.000508734 | 3.85340498 | 1.298123721 | 1.298123721 | 1 |
| PTPRT_20_40761966_40770575_40995945_41003669_FR | PTPRT | 0.390974701 | 0.390974701 | 5.898131356 | 0.000107101 | 0.002179292 | 1.336206195 | 1.31127902 | 1.31127902 | 1 |
| PTPRT_20_40695490_40704819_40853486_40862226_RF | PTPRT | 0.363092378 | 0.363092378 | 6.022994423 | 8.96E-05 | 0.001959674 | 1.521218337 | 1.286179837 | 1.286179837 | 1 |
| RAN_12_131315466_131318726_131332056_131334187_RR | RAN | 0.409645167 | 0.409645167 | 4.697687617 | 0.000668175 | 0.00665819 | -0.555916376 | 1.328359062 | 1.328359062 | 1 |
| RB1_13_48835536_48838517_49000831_49010576_FR | RB1 | -0.421233583 | -0.421233583 | -6.218242572 | 6.80E-05 | 0.001663343 | 1.806054195 | 0.746785809 | -1.339072045 | -1 |
| REL_2_61090704_61099366_61123363_61128146_FF | REL | -0.396733033 | -0.396733033 | -8.151247086 | 5.78E-06 | 0.000384323 | 4.347158457 | 0.759576389 | -1.316523281 | -1 |
| REL_2_61090704_61099366_61149976_61161058_FF | REL | -0.36693886 | -0.36693886 | -7.752686136 | 9.27E-06 | 0.000506975 | 3.862472978 | 0.775426067 | -1.289613597 | -1 |
| REL_2_61090704_61099366_61144132_61147262_FR | REL | 0.677200702 | 0.677200702 | 15.31760281 | 1.02E-08 | 7.96E-06 | 10.6128694 | 1.599034097 | 1.599034097 | 1 |
| RGS6_14_72418571_72425681_72679959_72689252_RR | RGS6 | 0.640246502 | 0.640246502 | 17.53962222 | 2.45E-09 | 2.49E-06 | 11.91593523 | 1.558595442 | 1.558595442 | 1 |
| ROR2_9_94323433_94326108_94448327_94455574_FF | ROR2 | -0.317797399 | -0.317797399 | -13.17593415 | 4.86E-08 | 1.71E-05 | 9.126251542 | 0.802293826 | -1.246426144 | -1 |
| SCGB1A1_11_62128712_62135211_62160970_62163465_FR | SCGB1A1 | -0.657694922 | -0.657694922 | -10.36431148 | 5.56E-07 | 8.40E-05 | 6.723090349 | 0.633890292 | -1.57756005 | -1 |
| SOS1_2_39227963_39230003_39353282_39361637_RF | SOS1 | 0.674466946 | 0.674466946 | 13.89064783 | 2.82E-08 | 1.38E-05 | 9.651002 | 1.596006963 | 1.596006963 | 1 |
| SOS1_2_39209340_39220780_39276526_39280091_FR | SOS1 | 0.461207721 | 0.461207721 | 10.99304586 | 3.08E-07 | 5.60E-05 | 7.313177343 | 1.376693805 | 1.376693805 | 1 |
| SRD5A2_2_31741633_31747723_31778586_31789876_FF | SRD5A2 | -0.437278492 | -0.437278492 | -17.05212796 | 3.30E-09 | 3.05E-06 | 11.6482057 | 0.738526456 | -1.354047634 | -1 |
| SRD5A2_2_31729027_31741633_31760980_31767977_FR | SRD5A2 | 0.394046044 | 0.394046044 | 9.411702288 | 1.44E-06 | 0.000162975 | 5.761374339 | 1.314073565 | 1.314073565 | 1 |
| SRD5A2_2_31760980_31767977_31778586_31789876_RF | SRD5A2 | 0.36636811 | 0.36636811 | 11.1293664 | 2.72E-07 | 5.28E-05 | 7.436768688 | 1.289103509 | 1.289103509 | 1 |
| TGFB2_1_218504155_218510817_218542394_218548723_RF | TGFB2 | -0.401060991 | -0.401060991 | -18.11165268 | 1.74E-09 | 2.22E-06 | 12.21825228 | 0.757301142 | -1.320478664 | -1 |
| TGFB2_1_218491029_218498929_218553354_218556593_FF | TGFB2 | -0.386196098 | -0.386196098 | -7.834000204 | 8.41E-06 | 0.000476069 | 3.962917896 | 0.765144376 | -1.306942888 | -1 |
| TMPRSS2_21_42841804_42850832_42927381_42930038_FR | TMPRSS2 | 0.46760546 | 0.46760546 | 8.609824216 | 3.43E-06 | 0.000272613 | 4.88179253 | 1.382812413 | 1.382812413 | 1 |
| TOP2A_17_38547618_38549511_38613131_38616534_RR | TOP2A | -0.334752665 | -0.334752665 | -8.436098305 | 4.17E-06 | 0.000304987 | 4.682072992 | 0.792920063 | -1.261161178 | -1 |
| TOP2A_17_38564762_38568693_38613131_38616534_RR | TOP2A | -0.33269637 | -0.33269637 | -9.020450371 | 2.18E-06 | 0.000221829 | 5.340676459 | 0.79405103 | -1.259364905 | -1 |
| TOP2B_3_25644985_25663188_25716096_25717154_FF | TOP2B | 0.677625777 | 0.677625777 | 11.54384037 | 1.88E-07 | 3.83E-05 | 7.803482889 | 1.599505304 | 1.599505304 | 1 |
| VEGFC_4_177629821_177639626_177693384_177697283_FR | VEGFC | 0.624813933 | 0.624813933 | 8.924483137 | 2.42E-06 | 0.00023044 | 5.235055692 | 1.542011936 | 1.542011936 | 1 |
| VEGFC_4_177629821_177639626_177740221_177743175_FR | VEGFC | 0.532875204 | 0.532875204 | 11.11732726 | 2.75E-07 | 5.28E-05 | 7.425914159 | 1.446809728 | 1.446809728 | 1 |
| VEGFC_4_177629821_177639626_177693384_177697283_FF | VEGFC | 0.418296493 | 0.418296493 | 13.22565823 | 4.68E-08 | 1.71E-05 | 9.163763582 | 1.336348688 | 1.336348688 | 1 |
| EZH2_7_148496931_148503515_148602692_148606606_FF | EZH2 | 0.221213903 | 0.221213903 | 6.686469603 | 3.59E-05 | 0.001181426 | 2.467211314 | 1.165714021 | 1.165714021 | 1 |
| EZH2_7_148496931_148503515_148610251_148614284_FR | EZH2 | 0.220468898 | 0.220468898 | 8.311487407 | 4.80E-06 | 0.000337006 | 4.53671325 | 1.165112204 | 1.165112204 | 1 |
| SP1_12_53752782_53754759_53771263_53775550_RF | SP1 | 0.197127842 | 0.197127842 | 4.00029596 | 0.002121214 | 0.014464632 | -1.742110313 | 1.146413768 | 1.146413768 | 1 |
| SP1_12_53771263_53775550_53824264_53827278_FR | SP1 | 0.193365041 | 0.193365041 | 3.676018582 | 0.003703744 | 0.02071572 | -2.31009962 | 1.143427617 | 1.143427617 | 1 |
| DAPK1_9_90064560_90073617_90176237_90180153_FF | DAPK1 | -0.210075392 | -0.210075392 | -5.311660617 | 0.000255365 | 0.00370395 | 0.437247364 | 0.864492054 | -1.156748631 | -1 |
| DAPK1_9_90064560_90073617_90339152_90340776_FF | DAPK1 | -0.289887636 | -0.289887636 | -3.250481175 | 0.007812935 | 0.034291978 | -3.064141545 | 0.817965763 | -1.222545056 | -1 |
| DAPK1_9_90064560_90073617_90140806_90142738_FR | DAPK1 | 0.299375751 | 0.299375751 | 7.197207444 | 1.84E-05 | 0.000805368 | 3.154114326 | 1.230611817 | 1.230611817 | 1 |
| DAPK1_9_90064560_90073617_90140806_90142738_FF | DAPK1 | 0.22308584 | 0.22308584 | 3.330633912 | 0.006781108 | 0.031049051 | -2.92175958 | 1.167227549 | 1.167227549 | 1 |
| FGD4_12_32760791_32767406_32781508_32786048_FR | FGD4 | -0.274580142 | -0.274580142 | -5.050237381 | 0.000382113 | 0.004709684 | 0.020720467 | 0.82669087 | -1.209642004 | -1 |
| FGD4_12_32760791_32767406_32781508_32786048_RR | FGD4 | -0.282349703 | -0.282349703 | -5.378249402 | 0.000230809 | 0.003536515 | 0.541798821 | 0.822250734 | -1.216174043 | -1 |
| FGD4_12_32714978_32722972_32735447_32738552_RR | FGD4 | 0.277743465 | 0.277743465 | 4.897130535 | 0.000486024 | 0.005488137 | -0.227642215 | 1.212297233 | 1.212297233 | 1 |
| FGD4_12_32714978_32722972_32768073_32772358_RR | FGD4 | 0.224649977 | 0.224649977 | 3.77524936 | 0.003119249 | 0.018634903 | -2.135456788 | 1.168493717 | 1.168493717 | 1 |
| GAB1_4_144235957_144242111_144369687_144374560_FF | GAB1 | 0.322283078 | 0.322283078 | 7.881995854 | 7.94E-06 | 0.000473286 | 4.021826263 | 1.250307607 | 1.250307607 | 1 |
| GAB1_4_144272552_144276220_144402622_144411096_FR | GAB1 | 0.232807271 | 0.232807271 | 5.862864002 | 0.000112692 | 0.002243704 | 1.283544618 | 1.175119334 | 1.175119334 | 1 |
| GAB1_4_144254752_144257034_144321110_144332903_RF | GAB1 | -0.1933323 | -0.1933323 | -3.299161865 | 0.00716858 | 0.03218585 | -2.977661138 | 0.874583297 | -1.143401668 | -1 |
| GAB1_4_144298156_144300750_144321110_144332903_FR | GAB1 | -0.203419346 | -0.203419346 | -6.533724514 | 4.41E-05 | 0.001301693 | 2.25489123 | 0.868489706 | -1.151424126 | -1 |
| HSD3B2_1_119937390_119948935_119959754_119963670_FR | HSD3B2 | 0.207425802 | 0.207425802 | 6.208022809 | 6.90E-05 | 0.001671497 | 1.791279777 | 1.154626147 | 1.154626147 | 1 |
| HSD3B2_1_119937390_119948935_119959754_119963670_FF | HSD3B2 | 0.167484698 | 0.167484698 | 5.70659508 | 0.000141491 | 0.002556892 | 1.048050136 | 1.123098683 | 1.123098683 | 1 |
| HSD3B2_1_119912462_119915175_119959754_119963670_RR | HSD3B2 | -0.168081632 | -0.168081632 | -3.274998031 | 0.007481356 | 0.033194645 | -3.020586815 | 0.890025372 | -1.123563476 | -1 |
| KLK2_19_51340459_51344004_51390533_51395187_FR | KLK2 | -0.233986179 | -0.233986179 | -5.085188859 | 0.000361865 | 0.004584902 | 0.076963796 | 0.850282306 | -1.176079983 | -1 |
| KLK2_19_51317027_51319938_51340459_51344004_FF | KLK2 | 0.259249519 | 0.259249519 | 3.256953029 | 0.007723978 | 0.033974819 | -3.052644106 | 1.196855945 | 1.196855945 | 1 |
| KLK2_19_51317027_51319938_51346270_51350944_FF | KLK2 | 0.251147882 | 0.251147882 | 3.297865414 | 0.007185018 | 0.032204968 | -2.979964123 | 1.190153686 | 1.190153686 | 1 |
| MAP3K14_17_43358197_43360790_43375304_43380378_FF | MAP3K14 | 0.227459078 | 0.227459078 | 4.562645956 | 0.000831476 | 0.00777086 | -0.781135175 | 1.170771132 | 1.170771132 | 1 |
| MAP3K14_17_43360790_43364282_43409961_43415408_FF | MAP3K14 | 0.198862372 | 0.198862372 | 4.316518127 | 0.00124648 | 0.010161606 | -1.197399861 | 1.147792913 | 1.147792913 | 1 |
| MAP3K14_17_43358197_43360790_43375304_43380378_RR | MAP3K14 | -0.205626917 | -0.205626917 | -6.208573083 | 6.89E-05 | 0.001671497 | 1.792075671 | 0.867161784 | -1.15318735 | -1 |
| MIF_22_24194490_24195811_24245843_24254074_RR | MIF | 0.254727783 | 0.254727783 | 10.76065239 | 3.82E-07 | 6.37E-05 | 7.098970594 | 1.193110598 | 1.193110598 | 1 |
| MIF_22_24206371_24208274_24245843_24254074_FR | MIF | 0.207093745 | 0.207093745 | 5.104189064 | 0.000351337 | 0.004526272 | 0.107467258 | 1.154360424 | 1.154360424 | 1 |
| MUC1_1_155176403_155179713_155191807_155193554_FR | MUC1 | -0.209333417 | -0.209333417 | -6.160281821 | 7.38E-05 | 0.001740993 | 1.722065484 | 0.864936775 | -1.156153871 | -1 |
| MUC1_1_155146523_155149986_155191807_155193554_FR | MUC1 | -0.218468452 | -0.218468452 | -5.993061073 | 9.35E-05 | 0.001993646 | 1.477069135 | 0.859477364 | -1.163497775 | -1 |
| RAD51_15_40972719_40979675_41025213_41027977_RF | RAD51 | 0.352545734 | 0.352545734 | 4.666716555 | 0.00070238 | 0.006893753 | -0.607362881 | 1.276811662 | 1.276811662 | 1 |
| RAD51_15_40937212_40938851_41025213_41027977_RF | RAD51 | -0.242505623 | -0.242505623 | -4.646371743 | 0.000725849 | 0.007053284 | -0.641225269 | 0.845275991 | -1.183045551 | -1 |
| RAD51_15_41009919_41011826_41025213_41027977_RF | RAD51 | -0.243901539 | -0.243901539 | -6.598967119 | 4.03E-05 | 0.001240188 | 2.34597507 | 0.844458518 | -1.184190791 | -1 |
| RNASEL_1_182541376_182556600_182605098_182607856_FR | RNASEL | 0.268497887 | 0.268497887 | 3.858975685 | 0.002700604 | 0.016960458 | -1.988638304 | 1.204553012 | 1.204553012 | 1 |
| RNASEL_1_182541376_182556600_182577916_182584530_FF | RNASEL | 0.25710383 | 0.25710383 | 3.235840558 | 0.008018044 | 0.03489118 | -3.090150814 | 1.195077211 | 1.195077211 | 1 |
| SRC_20_35928873_35935451_35989678_35993330_FR | SRC | 0.206516047 | 0.206516047 | 6.456694813 | 4.89E-05 | 0.001394478 | 2.146589494 | 1.153898277 | 1.153898277 | 1 |
| SRC_20_35928873_35935451_35989678_35993330_FF | SRC | 0.198581558 | 0.198581558 | 6.507686654 | 4.56E-05 | 0.001334586 | 2.218375228 | 1.147569522 | 1.147569522 | 1 |
| SRD5A3_4_56188038_56191526_56242301_56245314_RF | SRD5A3 | 0.266992266 | 0.266992266 | 4.835274287 | 0.000536131 | 0.005815136 | -0.328887879 | 1.203296575 | 1.203296575 | 1 |
| SRD5A3_4_56209429_56213336_56242301_56245314_RF | SRD5A3 | 0.239396914 | 0.239396914 | 4.842348143 | 0.000530134 | 0.005781472 | -0.317283393 | 1.180499078 | 1.180499078 | 1 |
| WNT1_12_49327866_49332429_49386082_49387249_RF | WNT1 | 0.171379721 | 0.171379721 | 4.015889993 | 0.002065728 | 0.014255673 | -1.715014756 | 1.126134949 | 1.126134949 | 1 |
| WNT1_12_49361168_49364315_49377006_49380965_RF | WNT1 | -0.188758659 | -0.188758659 | -6.377243699 | 5.46E-05 | 0.001476292 | 2.0340141 | 0.877360306 | -1.139782588 | -1 |
| WNT1_12_49327866_49332429_49364343_49365445_FF | WNT1 | -0.289012147 | -0.289012147 | -10.04098231 | 7.63E-07 | 9.95E-05 | 6.406185639 | 0.81846229 | -1.221803389 | -1 |
| Abbreviations. logFC: logarithm of the fold change; AveExpr: Average expression; adj.P·Val: Adjusted p-value; B: B-statistic (log-odds that that gene is differentially expressed); FC: Fold change; FC_1: Fold change centered around 1; Binary: Binary call for loop presence/absence. | | | | | | | | | | |

| Table S6. Markers for high-risk category 3 vs low-risk category 1 and for high-risk category 3 vs intermediate-risk category 2. | | | | | |
| --- | --- | --- | --- | --- | --- |
| **Biomarkers for high-risk category 3 vs low-risk category 1** | | | **Biomarkers for high-risk category 3 vs intermediate-risk category 2** | | |
| **Loci** | **Gene location** | **Markers** | **Loci** | **Gene location** | **Markers** |
| 6_7724582_7733496_7801590_7806316_FF | BMP6 | PCa.119.37.39 | 1_119912462_119915175_119959754_119963670_RR | HSD3B2 | PCa.119.129.131 |
| 21_39895678_39899145_39984806_39991905_RF | ERG | PCa.119.65.67 | 4_177629821_177639626_177740221_177743175_FR | VEGFC | PCa.119.205.207 |
| 8_16195878_16203315_16396849_16400398_FF | MSR1 | PCa.119.77.79 | 12_99061113_99062942_99098781_99108240_FF | APAF1 | PCa.119.49.51 |
| 1_155146523_155149986_155191807_155193554_FR^*^ | MUC1 | PCa.119.121.123 | 1_155146523_155149986_155191807_155193554_FR^*^ | MUC1 | PCa.119.121.123 |
| 11_107955219_107960166_108013361_108018367_FF^√^ | ACAT1 | PCa.119.57.59 | 11_107955219_107960166_108013361_108018367_FF^√^ | ACAT1 | PCa.119.57.59 |
| 9_90064560_90073617_90140806_90142738_FR^●^ | DAPK1 | PCa.119.165.167 | 9_90064560_90073617_90140806_90142738_FR^●^ | DAPK1 | PCa.119.165.167 |
| ^*, √, ●^ Last three markers are common.  Abbreviations. ACAT1: acetyl-CoA acetyltransferase 1; APAF1: apoptotic peptidase activating factor 1; BMP6: bone morphogenetic protein 6; DAPK1: death associated protein kinase 1; ERG: ETS transcription factor ERG; HSD3B2: hydroxy-delta-5-steroid dehydrogenase, 3 beta- and steroid delta-isomerase 2; MSR1: macrophage scavenger receptor 1; MUC1: mucin 1, cell surface associated; VEGFC: vascular endothelial growth factor C. | | | | | |

| Table S7.  Comparison of pathology and EpiSwitch™ results for category 3 vs 1 classifier. | | |
| --- | --- | --- |
|  | Pathology results | |
| EpiSwitch™ diagnosis | Category 1 | Category 3 |
| Category 1 | 39 | 5 |
| Category 3 | 3 | 20 |

| Results from classification of blinded samples for category 3 vs 1 classifier (n=67). | | |
| --- | --- | --- |
| **Statistic** | **Value** | **95% CI** |
| Sensitivity | 80.00% | 59.30% to 93.17% |
| Specificity | 92.86% | 80.52% to 98.50% |
| Positive Likelihood Ratio | 11.20 | 3.70 to 33.91 |
| Negative Likelihood Ratio | 0.22 | 0.10 to 0.47 |
| Disease prevalence (*) | 37.31% | 25.80% to 49.99% |
| Positive Predictive Value (*) | 86.96% | 68.77% to 95.28% |
| Negative Predictive Value (*) | 88.64% | 78.00% to 94.49% |
| (*) These values are dependent on disease prevalence.  Abbreviations. 95% CI: 95% confidence interval. | | |

| Table S8.  Comparison of pathology and EpiSwitch™ results for category 3 vs 2 classifier. | | |
| --- | --- | --- |
|  | Pathology results | |
| EpiSwitch™ diagnosis | Category 2 | Category 3 |
| Category 2 | 16 | 4 |
| Category 3 | 2 | 21 |

| Results from classification of blinded samples for category 3 vs 2 classifier (n=43). | | |
| --- | --- | --- |
| **Statistic** | **Value** | **95% CI** |
| Sensitivity | 84.00% | 63.92% to 95.46% |
| Specificity | 88.89% | 65.29% to 98.62% |
| Positive Likelihood Ratio | 7.56 | 2.02 to 28.24 |
| Negative Likelihood Ratio | 0.18 | 0.07 to 0.45 |
| Disease prevalence (*) | 58.14% | 42.13% to 72.99% |
| Positive Predictive Value (*) | 91.30% | 73.76% to 97.51% |
| Negative Predictive Value (*) | 80.00% | 61.62% to 90.88% |
| (*) These values are dependent on disease prevalence.  Abbreviations. 95% CI: 95% confidence interval. | | |
